# Site-Specific Trafficking of Lipid and Polar Metabolites in Adipose and Muscle Tissue Reveals the Impact of Bariatric Surgery-Induced Weight Loss: A 6-Month Follow-Up Study

**DOI:** 10.3390/metabo15080525

**Published:** 2025-08-02

**Authors:** Aidan Joblin-Mills, Zhanxuan E. Wu, Garth J. S. Cooper, Ivana R. Sequeira-Bisson, Jennifer L. Miles-Chan, Anne-Thea McGill, Sally D. Poppitt, Karl Fraser

**Affiliations:** 1 Food Chemistry and Structure, AgResearch Limited, Palmerston North 4442, New Zealand; ajoblinmills@gmail.com (A.J.-M.); emily.wu@agresearch.co.nz (Z.E.W.); karl.fraser@agresearch.co.nz (K.F.); 2 High-Value Nutrition National Science Challenge, Auckland 1010, New Zealand; i.sequeira@auckland.ac.nz (I.R.S.-B.); j.miles-chan@auckland.ac.nz (J.L.M.-C.); 3 School of Health Sciences, Massey University, Palmerston North 4442, New Zealand; 4 School of Biological Sciences, University of Auckland, Auckland 1010, New Zealand; g.cooper@auckland.ac.nz; 5 Centre for Advanced Discovery and Experimental Therapeutics, School of Medical Sciences, University of Manchester, Manchester M13 9NT, UK; 6 Riddet Institute, Massey University, Palmerston North 4442, New Zealand; 7 Human Nutrition Unit, School of Biological Sciences, University of Auckland, Auckland 1024, New Zealand; 8 Te Kāika Queenstown/Ōamaru, Ōtākou Health Limited, Caversham, Dunedin 9012, New Zealand; anne.thea.mcgill@gmail.com; 9 Department of Medicine, University of Auckland, Auckland 1010, New Zealand

**Keywords:** metabolomics, plasma, laparoscopic samples, multivariate statistics, amino acids, Jaccard–Tanimoto coefficients

## Abstract

Background: The causation of type 2 diabetes remains under debate, but evidence supports both abdominal lipid and ectopic lipid overspill into tissues including muscle as key. How these depots differentially alter cardiometabolic profile and change during body weight and fat loss is not known. Methods: Women with obesity scheduled to undergo bariatric surgery were assessed at baseline (BL, *n* = 28) and at 6-month follow-up (6m_FU, *n* = 26) after weight loss. Fasting plasma (Pla), subcutaneous thigh adipose (STA), subcutaneous abdominal adipose, (SAA), and thigh *vastus lateralis* muscle (VLM) samples were collected at BL through surgery and at 6m_FU using needle biopsy. An untargeted liquid chromatography mass spectrometry metabolomics platform was used. Pla and tissue-specific lipid and polar metabolite profiles were modelled as changes from BL and 6m_FU. Results: There was significant body weight (−24.5 kg) loss at 6m_FU (*p* < 0.05). BL vs. 6m_FU tissue metabolomics profiles showed the largest difference in lipid profiles in SAA tissue in response to surgery. Conversely, polar metabolites were more susceptible to change in STA and VLM. In Pla samples, both lipid and polar metabolite profiles showed significant differences between timepoints. Jaccard–Tanimoto coefficient t-tests identified a sub-group of gut microbiome and dietary-derived omega-3-fatty-acid-containing lipid species and core energy metabolism and adipose catabolism-associated polar metabolites that are trafficked between sample types in response to bariatric surgery. Conclusions: In this first report on channelling of lipids and polar metabolites to alternative tissues in bariatric-induced weight loss, adaptive shuttling of small molecules was identified, further promoting adipose processing and highlighting the dynamic and coordinated nature of post-surgical metabolic regulation.

## 1. Introduction

Differing body fat storage sites are known to present varying risks of adverse metabolic health, with higher visceral to subcutaneous adipose tissue commonly reflecting greater susceptibility to metabolic complications such as type 2 diabetes (T2D) and cardiovascular (CV) disease [1]. In addition to deposition of lipid into abdominal or visceral adipose, evidence points towards adipose dysfunction alongside lipid ‘overspill’ into ectopic non-adipose sites such as muscle, upper body intra-dermis, the liver, and pancreas as key drivers [2,3,4]. Bariatric surgery significantly decreases adipose mass and is a highly successful treatment for obesity, commonly resolving associated T2D [5]. The precise sites of lipid loss remain poorly defined however, in particular within ectopic sites, as are the metabolic sequelae. Procedures include sleeve (or partial) gastrectomy (SG) where decrease in stomach size and tubular shape inhibits gastric distension and food intake, and Roux-en-Y gastric bypass (RYGB), where a small stomach pouch is created and a Y-shaped section of the small intestine is attached to allow food to bypass the distal stomach, duodenum, and proximal jejunum [6]. Both methods can be performed via laparoscopy with minimal risk to patients and result in significant weight loss. Recent studies have shown that implementing a very low energy diet (VLED) in the weeks preceding bariatric surgery has several metabolic and clinical benefits in patients and can significantly reduce the size of the liver, facilitating surgical access to the stomach during the laparoscopic procedure. This initial VLED is particularly important in individuals with high visceral adiposity as it reduces operative time, lowers the risk of conversion to open surgery, and decreases intra-operative and post-operative complications [7,8].

Metabolomics is a powerful approach for profiling small-molecule signatures and monitoring biochemical changes across tissues and biofluids in participants and model organisms. Blood-based metabolomics, due to its minimally invasive nature, has been widely applied for the early diagnosis and prognosis of many metabolic diseases [9,10,11]. However, relying solely on circulating biomarkers offers limited insights into tissue-specific metabolic dysfunctions, which are often more directly implicated in disease pathogenesis [12,13,14]. Evidence indicates that adipose depots and skeletal muscle tissues possess distinct metabolic phenotypes and exhibit differential responses to interventions such as weight loss or dietary modification [15,16]. In a cohort of women with long-term overweight or obesity, we previously characterised the metabolome of multiple adipose tissue and skeletal muscle and liver sites [17,18], showing plasma metabolite and lipidome profiles to both best represent the liver profile and underscoring the importance of investigating individual tissue biomarker profiles [18]. Most studies have employed a targeted metabolomics approach to characterising metabolic status or disease prognosis, often focusing on specific metabolite classes such as sphingolipids, acylcarnitines, and BCAAs [15,19,20]. Although these are reliable biomarkers with ontological annotations, this reduced scope of metabolomics ignores many small molecule changes that work in tandem or provide novel mechanisms of action [21,22].

In addition, the novel concept of metabolite trafficking, whereby lipids and polar metabolites are actively shuttled between compartments, has garnered interest as a potential mechanism underlying systemic metabolic adaptation [23]. However, direct measurement for such coordinated metabolic rerouting remains scarce. More recent studies have captured the dynamic exchange of lipidomic information between different tissues and sites of interest utilising the Jaccard–Tanimoto similarity coefficient (JTC) in a novel non-parametric approach to determine switching of lipid metabolites within an established biological network [24,25]. While these studies have successfully shown the utility of the JTC for inferring the switching of lipids between compartments, the application of JTC for network trafficking frameworks has yet to be applied using polar metabolites or to human clinical intervention studies.

Therefore, the primary aim of this study was to address this gap by characterising the systemic metabolomic (lipid and polar metabolite) trafficking network and elucidating inter-compartmental relationships. We specifically explored whether patterns of metabolite abundance between tissues suggest inter-tissue communication and how these may be altered in response to aggressive weight loss interventions via bariatric surgery procedures. Using untargeted liquid chromatography–mass spectrometry (LC–MS) and feature traffic analysis based on Jaccard–Tanimoto similarity indices, here, we characterise both abundance changes and the inter-tissue movement of metabolites. Our aim is to identify distinct metabolic signatures associated with surgical body weight and fat mass loss and uncover the mechanisms by which metabolic reprogramming may occur across tissues. This approach provides critical insights into the spatial and dynamic complexity of human metabolism and establishes a foundation for future biomarker development and therapeutic targeting in obesity-related diseases.

## 2. Materials and Methods

### 2.1. Study Participants and Procedures

A cohort of *n* = 28 women with long-term overweight and obesity who were resident in the Auckland region was recruited for this 6-month follow-up (6m_FU) study. Inclusion criteria were women aged 18–60 years with a body mass index (BMI) > 40 kg/m^2^ or BMI > 35 kg/m^2^ with co-morbidities and who were eligible for surgery. All participants were scheduled for weight loss through laparoscopic bariatric surgery. Procedures were either sleeve gastrectomy (SG, *n* = 11) or Roux-en-Y gastric bypass (RYGB, *n* = 17; Supplementary Table S1). Prior to bariatric surgery all participants were enrolled into the trial and completed a 2–6-week total meal replacement diet using a commercial VLED (2.4 MJ/day intake, Optifast, Nestlé, Vevey, Switzerland) programme, as per New Zealand bariatric surgery guidelines, with the intent of decreasing the physical size of the liver through the promotion of hepatic lipid oxidation and glycogenolysis, thereby making the surgical procedure easier. Recent meta-analyses have also shown improved post-operative morbidity [8]. There were 3 clinical investigation days (CIDs) during the study: enrolment (Enrol), baseline (BL), and 6m_FU. The VLED programme began at CID1/Enrol and was completed prior to CID2/BL surgery.

At CID1/Enrol, demographic information, anthropometry (height, body weight, and waist circumference), and a venous blood sample (Plasma, Pla) were collected. Due to four participants beginning the VLED programme prematurely, their anthropometry and Pla data was not obtained for the CID1/Enrol timepoint. At CID2/baseline (BL), anthropometry (height and body weight) and body composition (total body fat and lean mass, measured supine, iDXA, GE Healthcare, WI, USA) were measured, and Pla and tissue samples were collected during the surgical procedure. Tissue samples comprised 7 samples of 3 different tissue types (adipose × 4, muscle × 2, and liver × 1). The adipose samples were collected from 4 tissue depots, 2 of which were considered to be ‘safe’ peripheral depots (subcutaneous thigh adipose, STA; subcutaneous abdominal adipose, SAA, collected above the hip), and 2 were ‘high-risk’ central depots (deep subcutaneous abdominal adipose, DSAA; intra-abdominal adipose, IAA). The muscle samples were collected from 2 sites: the thigh (*vastus lateralis* muscle, VLM) and abdomen (*rectus abdominis* muscle, RAM). STA and VLM samples were collected from a single trocar cut of the thigh, with the STA sample superficial relative to the deeper VLM sample. The liver sample was collected from 1 site (wedge of the right lobe). BL outcomes from untargeted metabolomics analyses of plasma and all 7 tissues have been published previously [18]. CID3/6m_FU was conducted in the clinic, with minor invasive surgical procedures. Pla was again collected, in addition to tissues samples that were accessible via subcutaneous dissection (adipose) or using a 5 mm diameter Bergstrom needle biopsy procedure (muscle) under local anaesthetic. These comprised 2 peripheral adipose (thigh STA, abdomen SAA) and 1 thigh muscle sample (VLM) only. The 3 assessment days (Enrol, BL surgery, and 6m_FU needle biopsy) were conducted after an overnight fast, and hence, all blood and tissue samples were collected in a fasted state. Outcomes from sample types (Pla, 2 × adipose, 1 × muscle) collected at Enrol, BL, and at 6m_FU are presented in this analysis (Table 1); the sample numbers utilised in the study’s statistical analyses are outlined unless stated otherwise. Participants were recruited at Auckland City Hospital, Auckland, New Zealand. Informed written consent was obtained at the Greenlane Clinical Centre, Auckland, or the University of Auckland Human Nutrition Unit, Auckland, New Zealand (ethics approval #NTX/08/10/103; trial registration—ACTRN12611000525987).

### 2.2. Materials

All organic solvents for metabolomics analysis (chloroform, methanol, acetonitrile, isopropanol, and formic acid) were obtained from Thermo Fisher Scientific (Auckland, New Zealand) and were of LC–MS grade except chloroform, which was of analytical grade. Milli-Q^®^ ultrapure water was obtained from Merck Millipore (Bedford, MA, USA). Ammonium formate (Fluka™, HPLC grade) was obtained from Sigma-Aldrich (Auckland, New Zealand). Lipid internal standard 1-palmitoyl(D_31_)-2-oleoyl-sn-glycero-3-phosphoethanolamine (16:0 d_31_-18:1-PE) was obtained from Avanti^®^ (Avanti Polar Lipids, Alabaster, AL, USA) and stored at −20 °C. Pre-dissolved polar metabolite internal standards (d_5_-L-tryptophan, d_4_-citric acid, d_10_-leucine, d_2_-tyrosine, d_35_-stearic acid, d_5_-benzoic acid, ^13^C-glucose, and d_7_-alanine) were obtained from Merck Millipore (Bedford, MA, USA) and stored at −20 °C.

### 2.3. Metabolomics Analyses

Metabolomics extractions were performed with 100 µL plasma or 25 mg frozen tissue using a bi-phasic extraction method previously described in [17]. All tissue biopsy and plasma sample processing was performed as outlined previously [18]. In brief, after tissues were homogenised in 800 µL pre-chilled extraction solvent, 400 µL H_2_O was added, followed by vortex mixing and centrifugation (11,000 rpm, 4 °C, 10 min). Plasma samples were mixed with 800 µL pre-chilled extraction solvent, vortex mixed, and stored at −20 °C for 60 min to allow protein precipitation; then, 400 µL H_2_O was added, followed by vortex mixing and centrifugation (11,000 rpm, 4 °C, 10 min). Finally, 200 µL of the upper aqueous layer and 200 µL of the bottom organic layer were transferred into separate 2 mL microcentrifuge tubes, evaporated under nitrogen, and stored at −80 °C until analysis. All remaining upper phases or lower phases from samples were pooled, respectively, for each sample matrix, aliquoted into 200 µL, and evaporated for QC samples. Blank samples were prepared following the same protocol with the absence of samples.

On the day of analysis, dried extracts were reconstituted and analysed as previously outlined [17,18]. Briefly, reconstituted polar metabolite extracts were separated on a SeQuant^®^ ZIC^®^-pHILIC 5 µm, 2.1 mm × 100 mm column (Merck, Darmstadt, Germany) and analysed using a Thermo LC–MS system consisting of an Accela 1250 quaternary UHPLC pump coupled to an Exactive Orbitrap mass spectrometer (Thermo Fisher Scientific, Waltham, MA, USA). Samples were analysed in both positive (ESI+) and negative electrospray ionisation (ESI-). Reconstituted lipid extracts were separated on an Acquity CSH™ C18 column 1.7 µm, 2.1 mm × 100 mm (Waters, Milford, MA, USA), and analysed using a Thermo LC–MS system comprising an Accela 1250 quaternary UHPLC system coupled to a Q Exactive hybrid quadrupole-Orbitrap mass spectrometer (Thermo Fisher Scientific, Waltham, MA, USA) using both ESI+ and ESI− modes.

Raw datafiles were converted to mzXML format with ProteoWizard (v 3.0.1818 [26]) and pre-processed with XCMS (v3.0.2) in R Studio (v3.2.2) [27]. Features were processed for alignment across all sample types and then divided into sample subsets to be normalised, respectively, with the appropriate pooled QC sample in the W4M Galaxy environment [28]. Blank features were filtered out via the XCMS diffreport function, and non-reproducible features were filtered through a CV < 30% calculation in QC samples. Lipids were annotated using LipidSearch software v4.1.16 on representative MS^2^ datafiles (Thermo Fisher Scientific, USA). Polar metabolites were annotated using an in-house (AgResearch) library based on authentic standards previously validated through HILIC LC–MS conditions identical to the current study. Unidentified features were searched against the HMDB (http://www.hmdb.ca/, accessed on 29 June 2025) and Lipid Maps (http://www.lipidmaps.org/, accessed on 29 June 2025) databases using a m/z mass error cutoff of less than 15 ppm for polar metabolite features or 10 ppm for lipid features.

After features were annotated, those without a confident identification were removed, and all adduct replicates between ESI+ and ESI− for a given metabolite, along with detected metabolite fragment products, were removed. The final resulting lipid or polar metabolite data matrix contained only unique representative annotations for each given lipid or polar metabolite feature. In total, the Enrol, BL, and 6m_FU lipidomics data matrix comprised 368 unique lipid features, with Pla samples having 199 lipid features, SAA tissues having 184 lipid features, STA tissues having 168 lipid features, and VLM tissues having 116 lipid features for statistical analysis. Similarly, the polar metabolomics data matrix comprised 128 unique polar metabolite features, with Pla samples having 85 polar metabolite features, SAA tissues having 184 polar metabolite features, STA tissues having 168 polar metabolite features, and VLM tissues having 116 polar metabolite features for statistical analysis.

### 2.4. Statistical Analyses

#### 2.4.1. Multivariate Statistics

Multivariate analysis was performed in SIMCA software version 18 (Sartorius, Umeå, Sweden). Unsupervised principal component analysis (PCA) was used to visualise Pla, SAA, STA, and VLM sample groups’ lipidome and polar metabolome profiles, depicted in group variation and relative group segregation from participants’ features. Supervised partial least squares discriminative analysis (PLS-DA) modelling was implemented to evaluate the change in all lipid and polar metabolite profiles within Pla, SAA, STA, and VLM sample groups, modelling feature changes for time of bariatric surgery (BL) relative to 6 months after bariatric surgery (6m_FU), but this also included time of enrolment (Enrol) relative to BL from the available Pla samples. Each initial sample-specific PLS-DA model’s features were projected to a normal probability distribution plot, and the top 30 variables of importance (VIP) discriminating between timepoints were isolated and re-projected to produce each model’s plots and cumulative metrics of variance (R2cum) and prediction (Q2cum). Models were subject to cross-validation–analysis of variance (CV-ANOVA) to validate each model’s significance (*p* ≤ 0.001).

#### 2.4.2. Univariate Statistics

All univariate statistics were calculated using R Studio (v12.1). BCAAs, including Valine (Val), Isoleucine (Ile), and Leucine (Leu), and aromatic amino acids (AAAs) Phenylalanine (Phe) and Tyrosine (Tyr) had their normalised peak intensity values isolated from the processed polar metabolite data matrix of Pla samples at CID1 (Enrol), CID2 (BL), and CID3 (6m_FU) for log2 fold change (FC) calculations. Additional FC comparisons for SAA, STA, and VLM sample groups at BL and 6m_FU were calculated. Paired t-tests were used to calculate the significance of each between-timepoint FC calculation, and each amino acid’s log10 normalised intensity values were presented in boxplots, showing each sample group’s levels at relative CIDs.

### 2.5. Jaccard Similarity Traffic Analysis

The Jaccard similarity traffic analysis evaluates sample sets matched by participants’ IDs, so a final analysis was performed on modified numbers of samples from Table 1, specifically 52 Pla samples (BL = 26 vs. 6m_FU = 26), 26 SAA samples (BL = 13 vs. 6m_FU = 13), 34 STA samples (BL = 17 vs. 6m_FU = 17), and 30 VLM samples (BL = 15 vs. 6m_FU = 15). All traffic analysis calculations were performed within Python 3.7.1 with the LipidTA package using windows PowerShell. Jaccard–Tanimoto coefficients (JTC*s*) were used as a non-parametric measure of the differential distribution of lipid and polar metabolite variables between Pla, SAA, STA, and VLM tissue compartments relative to our 6-month bariatric surgery follow-up model (BL vs. 6m_FU) [29,30]. The lipid (LTA) and polar metabolite (MTA) input matrices had their features signal corrected (divided by the sum of signals for that sample) in order to compare features across the dynamic range of each sample group’s relative concentrations. Furthermore, features were subject to an additional filtering step wherein value intensities were regarded as sufficient if they had a signal strength > 0 in ≥50% of samples in either CID group. The switch-associated *p*-values were calculated following the method of Rahman et al. [31] wherein the denoted *p*-value associated with each JTC represents the probability that the difference between the lists of variables for the timepoint phenotypes occurred by random chance irrespective of the traditional Student’s *t*-test *p*-value. To determine the significance of traffic analysis abundance changes, LipidTA embedded a Student’s *t*-test analysis with a corrected *p*-value of 0.0021, as previously calculated using the LipidTA developer’s findings [32]. For each comparison, the detection of signal data resulted in either FALSE, depicting no feature signal, or TRUE, depicting an abundance of feature signal above a threshold (threshold = 0.3). Features shown to switch between compartments with time (BL vs. 6m_FU) were plotted for a visual evaluation of the network diagrams and subsequently categorised into types of feature switches.

## 3. Results

### 3.1. Diet and Bariatric-Induced Body Weight and Composition Changes

A cohort of 28 women with obesity was enrolled into this 6-month bariatric surgery follow-up study. The mean (SD) age of the cohort was 43.9 (8.1) years, the mean body weight was 115.4 (17.3) kg, and the mean BMI was 42.8 (5.7) kg/m^2^. At enrolment, 24 Pla samples were collected for CID1 (Enrol), with four participants omitted due to hospital scheduling of an early start of the preoperative VLED weight loss programme before sample collection. The VLED programme resulted in a significant (*p* < 0.05) mean (SD) weight loss of −9.2 (4.2) kg over 2–6 weeks. At bariatric surgery CID2 (BL), Pla and multiple tissue samples were collected from 28 participants. One woman withdrew from the study following the bariatric procedure, one woman was excluded at 6m_FU with widespread infected eczema; hence, twenty-six women underwent the CID3 (6m_FU) assessments, including Pla, and subcutaneous adipose and Bergstrom needle biopsy tissue collection. As expected, following surgery, the mean body weight significantly decreased by −24.5 kg (*n* = 26, −21.2%) to 90.9 (13.3) kg, and the mean BMI also decreased by −9.2 kg/m^2^ to 33.6 (4.6) kg/m^2^ over 6 months post-surgery (both, *p* < 0.05). There was no significant difference in body weight loss between the two bariatric procedures (*p* > 0.05). More details on participant ethnicity, anthropometry, and surgical procedures can be found in the supplementary material (Supplementary Table S1).

### 3.2. Multivariate Metabolomic Profiling of Diet and Bariatric Surgery-Induced Weight Loss Shows Differences in Plasma and Tissue Responses

PCA modelling of the combined BL and 6m_FU Pla, SAA, STA, and VLM lipid profiles show clear segregation of Pla features from the three tissue biopsies (SAA, STA, and VLM) (Supplementary Figure S1a). In comparison, the polar metabolite profiles show clear segregation between all four sample types, with reduced variational spread amongst Pla, SAA, and STA samples, and an increased spread of variation amongst the VLM samples of polar metabolite profiles (Supplementary Figure S1b).

**Figure 1:**
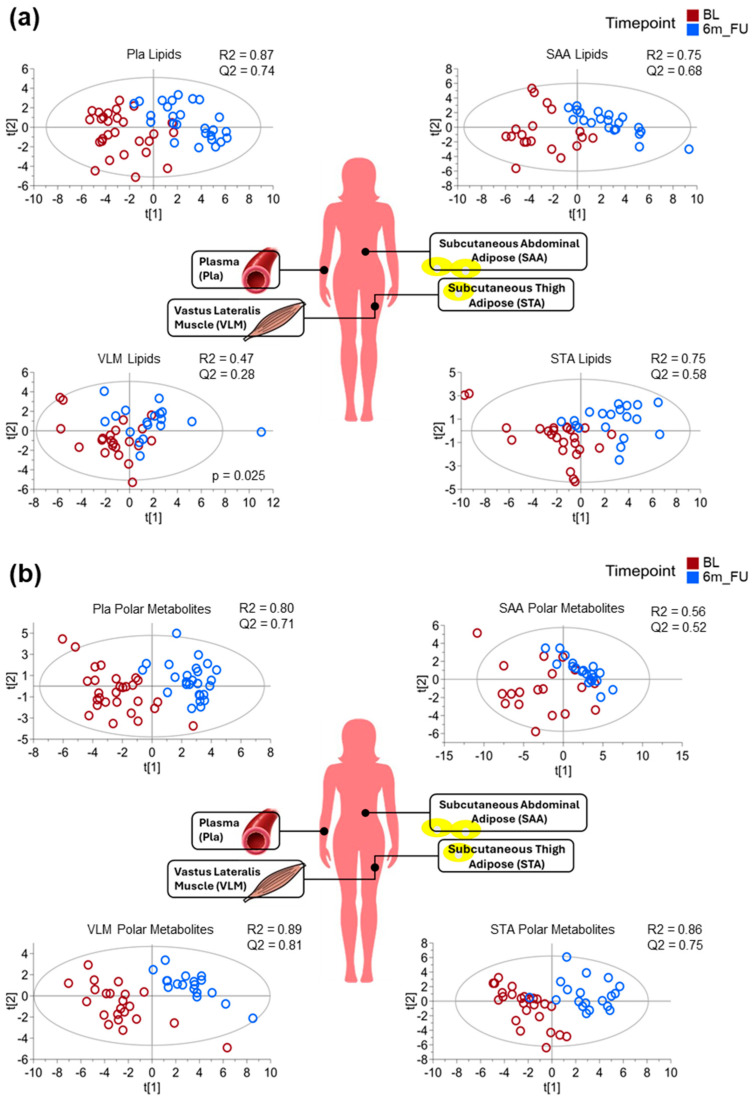
Top VIP features partial least squares discrimination analysis (PLS-DA) plots showing separation of (**a**) lipid profiles and (**b**) polar metabolite profiles at CID2 baseline, time of surgery (BL, red) vs. CID3 6-month post-surgery follow-up (6m_FU, blue) in plasma (Pla), subcutaneous abdominal adipose (SAA), subcutaneous thigh adipose (STA), and *vastus lateralis* muscle (VLM). Models present respective cumulative metrics of variance (R2cum) and prediction (Q2cum). Models were determined significant (*p* ≤ 0.001) by cross validation (CV) ANOVA, unless indicated otherwise.

PLS-DA modelling of the Pla and tissue-specific lipid and polar metabolite profile changes associated with BL vs. 6m_FU reveal differing degrees of sample responses to the cohort’s surgical procedures (Figure 1a,b). PLS-DA modelling significantly (*p* < 0.01) discriminated with strong prediction (Q2 ≥ 0.67) the lipid profile changes associated with bariatric surgery within the Pla and SAA samples. While the STA bariatric follow-up lipid profile model was deemed significant via CV-ANOVA, the predictive metric was only of moderate strength (Q2 = 0.58). The VLM bariatric follow-up lipid model had a weak predictive metric and was deemed insignificant (Q2 < 0.33, *p* = 0.025). For polar metabolite models, all were significant (*p* < 0.01), but the strength in prediction metrics contrasted with the lipid models, with VLM polar metabolites resulting in the strongest prediction metric (Q2 = 81) between BL and 6m_FU, followed by STA samples (Q2 = 75) and then Pla samples (Q2 = 0.71). The SAA bariatric follow-up polar metabolites model was only moderately strong (Q2 = 0.52).

PLS-DA was also used to model changes in Enrol vs. BL Pla. Although it showed less of a response than lipid and polar metabolite models in BL vs. 6m_FU Pla, it still calculated a strong separation of both lipids (Q2 = 0.71) and polar metabolites (Q2 = 0.69) during the 2–6-weeks of VLED weight loss (Supplementary Figure S2). The annotated features contributing to the lipid and polar metabolite model’s CID separation metrics, from each plasma and tissue biopsy, can be found in the supplementary material (Supplementary Tables S2–S9).

### 3.3. Analysis of Branched Chain and Aromatic Amino Acid Levels Within Plasma and Tissue Biopsies in Response to Bariatric Surgery Procedures

**Figure 2:**
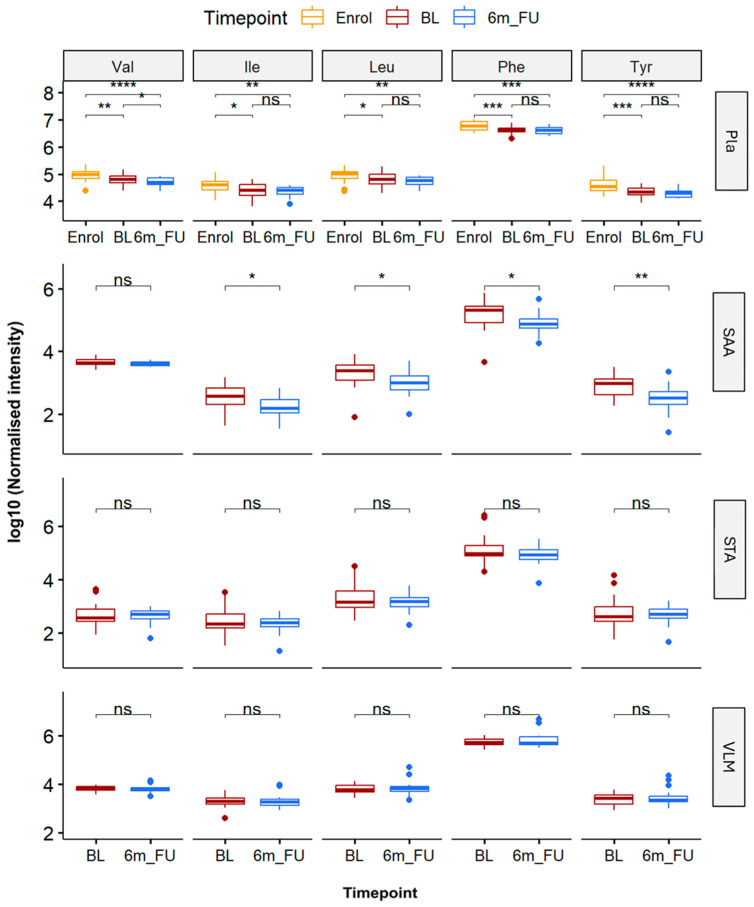
Boxplots showing fold change (log10) of branched-chain amino acids (BCAAs) Valine (Val), Isoleucine (Ile), and Leucine (Leu) and aromatic amino acids (AAAs) Phenylalanine (Phe) and Tyrosine (Tyr) from participants’ samples at CID1 enrolment, prior to start of very-low-energy diet (VLED) weight loss programme (Enrol, yellow); at CID2 baseline, time of surgery (BL, red); and at CID3 6-month post-surgery follow-up (6m_FU, blue). Subsequent boxplots compare amino acid levels at BL vs. 6m_FU for subcutaneous abdominal adipose (SAA), subcutaneous thigh adipose (STA), and *vastus lateralis* muscle (VLM) tissue samples. Student’s t-tests indicate log2 fold change significance of ns, *p* > 0.05; * *p* ≤ 0.05; ** *p* ≤ 0.01; *** *p* ≤ 0.001, **** *p* ≤ 0.0001.

As previous studies highlighted the importance of some key amino acids during weight loss, we investigated whether the levels of Valine (Val), Isoleucine (Ile), and Leucine (Leu) BCAAs and Phenylalanine (Phe) and Tyrosine (Tyr) AAAs changed in Pla and tissue biopsy (SAA, STA, and VLM) samples in response to aggressive weight loss (Figure 2).

We also included Pla samples collected at CID1 (Enrol) in the amino acid analysis; hence, there were three timepoints analysed (Enrol, BL, and 6m_FU). Student’s t-test calculations and the log2 fold change (FC) analysis of target BCAAs and AAAs in Pla showed a significant reduction (*p* < 0.01) in Val (FC = −0.23), Ile (FC = −0.22), Leu (FC = −0.21), Phe (FC = −0.22), and Tyr (FC = −0.36) due to the VLED weight loss programme (i.e., Enrol vs. BL). Although a phenotypic reduction in BCAA and AAA levels was sustained (*p* < 0.05) over the course of both Enrol vs. BL and Enrol vs. 6m_FU, only Val showed a significant FC reduction (FC = −0.14, *p* = 0.04) in Pla during the 6-month bariatric surgery follow-up analysis (BL vs. 6m_FU). Surprisingly, of the three tissue biopsies (SAA, STA, and VLM), only the SAA tissue samples showed a significant (*p* < 0.05) FC reduction in amino acids Ile (FC = −0.43), Leu (FC = −0.43), Phe (FC = −0.39), and Tyr (FC = −0.59) during the 6-month bariatric surgery follow-up (BL vs. 6m_FU).

### 3.4. Jaccard–Tanimoto Traffic Analysis of Lipid and Polar Metabolite Changes Between Sites Identifies Changes Due to Bariatric Surgery Induced Weight Loss

**Figure 3:**
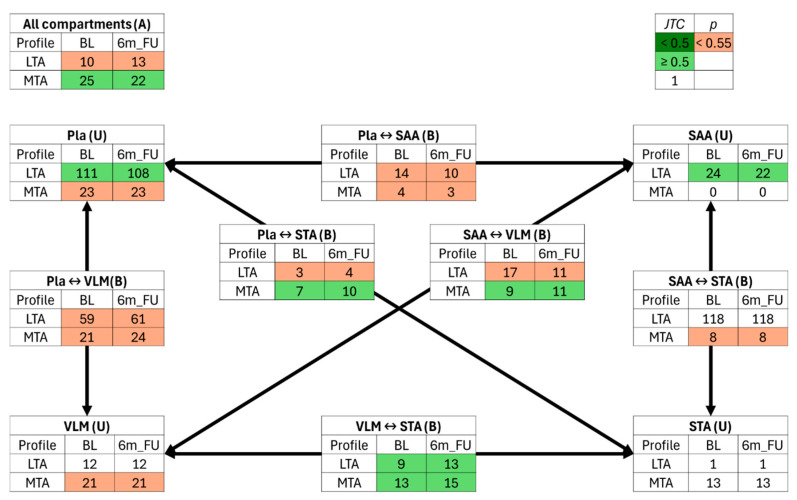
Lipid traffic analysis (LTA) and polar metabolite traffic analysis (MTA) calculate the change in feature abundance between compartments due to surgery-induced weight loss (BL vs. 6m_FU). Plasma (Pla), subcutaneous abdominal adipose (SAA), subcutaneous thigh adipose (STA) and *vastus lateralis* muscle (VLM). A-type features are switches found within all compartments (A), B-type features are switches found between two compartments (B), and U-type features are switches unique to one compartment (U). Jaccard–Tanimoto coefficients (JTC*s*) < 0.5 (dark green) indicate that less than 50% of features are in both groups, whereas JTC > 0.5 (light green) indicates that more than 50% features are in both groups. If JTC = 1 (white), all features are in both groups. All JTC values with a *p*-value < 0.55 are coloured orange, indicating that at least one of the features in each group does not appear in the other. If *p* = 1, then all features in one group are the same in the other, while *p* = 0 indicates that no features are the same in both groups.

The previously developed network traffic/switch analysis utilising the Jaccard–Tanimoto coefficient (JTC) of similarity was implemented here to identify the lipids (lipid traffic analysis, LTA) and polar metabolites (metabolite traffic analysis, MTA) that are above and below the limit of detection in a given compartment (U type), between adjacent compartments (B type, e.g., VLM ↔ STA), or ubiquitously throughout all samples (A type) (Figure 3).

Switch analysis of the lipid and polar metabolite signals amongst Pla and tissue samples highlighted distinct tissue characteristics that were reflected in the PLS-DA results. For instance, no change in U-type lipid activity in VLM was detected, while VLM polar metabolites showed a significant (*p* < 0.55, orange coloured) change in activity between BL and 6m_FU. Similarly, there was no significant change in polar metabolite activity within SAA, but there was a significant change in U-type lipids.

**Figure 4:**
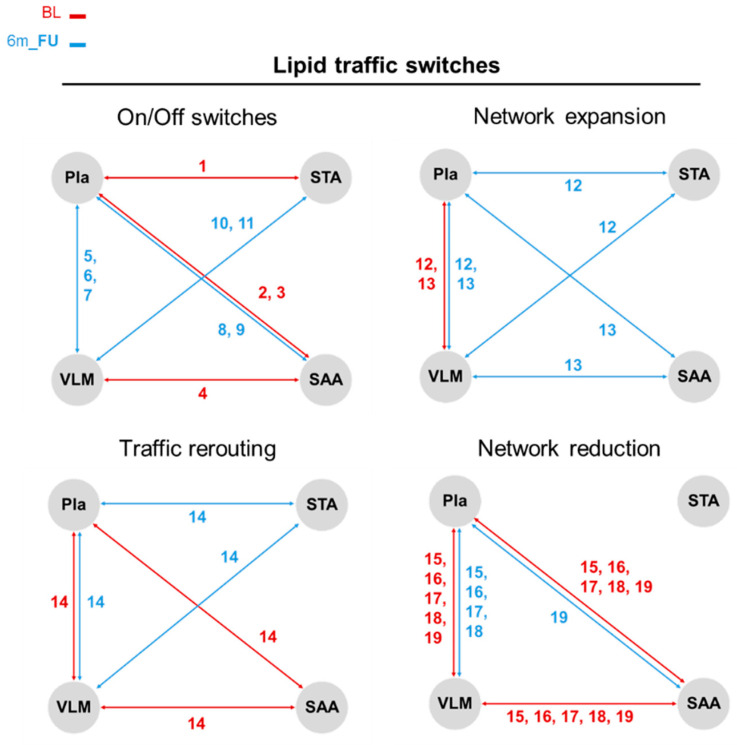
Lipid traffic analysis (LTA) identifies four types of network signalling switches that characterise the change in feature flow between compartments due to bariatric surgery-induced weight loss. Plasma (Pla), subcutaneous abdominal adipose (SAA), subcutaneous thigh adipose (STA), and *vastus lateralis* muscle (VLM). Baseline (BL, red) and 6-month follow-up (6m_FU, blue). Coloured numbers correspond to lipids annotated in Table 2.

**Figure 5:**
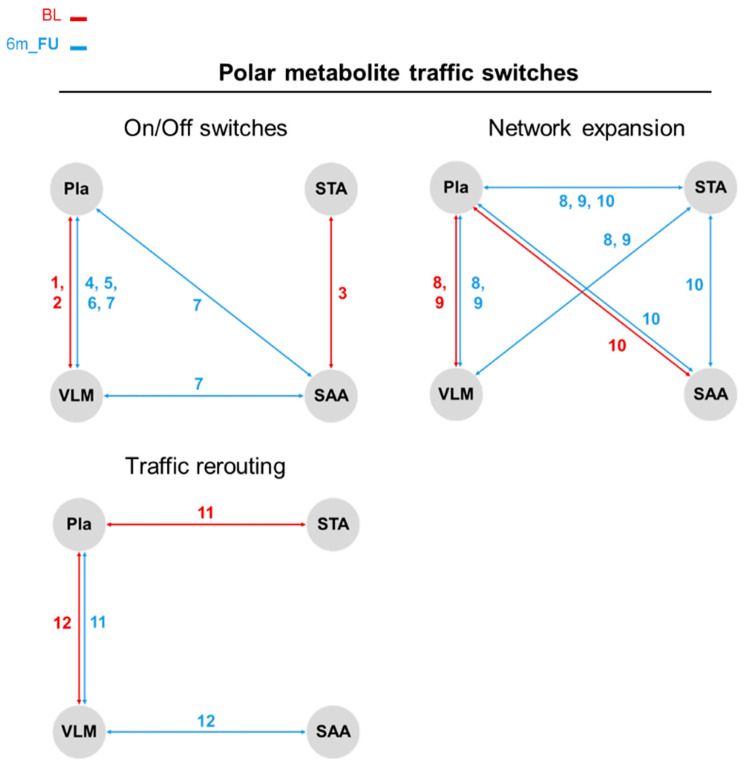
Polar metabolite traffic analysis (MTA) identifies three types of network signalling switches that characterise the change in feature flow between compartments due to surgery-induced weight loss. Plasma (Pla), subcutaneous abdominal adipose (SAA), subcutaneous thigh adipose (STA), and *vastus lateralis* muscle (VLM). Baseline (BL, red) and 6-month follow-up (6m_FU, blue). Coloured numbers correspond to polar metabolites annotated in Table 3.

Interestingly, our Jaccard–Tanimoto coefficients depicted no change (*J* = 1, white coloured) in both lipid and polar metabolite features within STA. Furthermore, there was no detectable trafficking of lipid signals between (B type) adipose samples (SAA ↔ STA), but there was a detectable trafficking of B-type polar metabolites, specifically Guanine and α-Aminoadipic acid (Figure 4 and Figure 5, Table 2 and Table 3). The most switch activity was detected within Pla samples and its tissue compartment pairings. Both Pla lipid and polar metabolite models indicated B-type switches for SAA and VLM compartment pairings, respectively, while only lipids, specifically SM(d16:0/25:1), PE(18:0p/20:4), and PI(16:0/20:4), were identified as B-type switches between Pla ↔ STA. There was a decrease (*J* = ≥ 0.5 and *p* ≥ 0.55, green coloured) in U-type lipid features within the Pla (−3) and SAA (−2) and an increase in B-type lipids trafficked between the thigh muscle and adipose tissues (VLM ↔ STA, +4). Interestingly, there was also an increase in B-type polar metabolites trafficked between the thigh tissues STA and VLM (+2). Apart from a global decrease (−3) in A-type polar metabolites shared between all sample types, an increase in B-type polar metabolites was also detected in both the Pla ↔ STA (+3) and VLM ↔ SAA (+2) compartment pairings. 

After further investigation into the discrete trafficking of features resulting from the LipidTA analysis tool with the inclusion of polar metabolites, we found that the movement of certain features between compartments from BL to 6m_FU fall into four types of switching categories, namely on/off switches, network expansion switches, compartment re-routing switches, and network reduction switches. 

In total, we annotated 19 lipids and 12 polar metabolite features that fit into these switching categories (Figure 4 and Figure 5; Table 2 and Table 3). Most of the lipids were found to be either trafficked between two compartments at BL and then lost in signal at 6m_FU (SM(d16:0/25:1), SM(d18:2/16:0), SM(d17:1/16:0), and PE(16:0p/18:2)) or vice versa (PC(16:0e/22:6), PE(18:0/20:3), SM(d20:0/18:1), SM(d16:1/16:0), SM(d22:1/16:0), PE(16:0p/20:5), and PG(18:1/18:1)), comprising the on/off switching category. Two lipids (PE(18:0p/20:4) and PC(16:0/20:5)), were classified under the category of network expansion, whereas at BL, they both trafficked between the Pla and VLM, and at 6m_FU, they expanded their compartment trafficking network to incorporate the STA or SAA compartments, respectively. Interestingly, the PI(16:0/20:4) lipid at BL was trafficked between Pla, VLM, and SAA compartments, but at 6m_FU began trafficking between the Pla, VLM, and the STA compartments instead, indicating a compartment rerouting type switch. Unsurprisingly, the remaining lipids fit into the lipid only category of network reduction, whereas lipids that were found to traffic between the Pla, VLM, and SAA compartments at BL lost signal trafficking routes between the VLM and SAA compartments at 6m_FU and only moved between the Pla and VLM compartments (PC(18:0/22:6), SM(d16:0/24:2), SM(d16:0/27:2), and SM(d18:1/18:0)) or between the Pla and SAA compartments (PC(18:0/18:1)).

For the polar metabolite switching categories, Acetylcarnosine, Guanidoacetic acid, and Guanine lost trafficking signals between compartments by 6m_FU, while 4-Imidazolone-5-propionic acid, Pyroglutamic acid, Trimethylamine N-oxide (TMAO), and 2-Aminooctanoic acid all gained trafficking signals between compartments at 6m_FU (i.e., on/off switches). While Guanine was found to move between STA and SAA, the remaining metabolites trafficked between Pla and VLM, with 2-Aminooctanoic acid additionally moving between SAA and Pla ↔ VLM. Network expansion type metabolites showed asymmetric dimethylarginine and N6,N6,N6-Trimethyl-L-lysine moving between Pla and VLM at BL, but at 6m_FU, they expanded to Pla ↔ VLM ↔ STA. Aminoadipic acid trafficking between Pla and SAA at BL expanded to Pla ↔ SAA ↔ STA at 6m_FU. The last switching type categorised for polar metabolites was network rerouting, wherein Methionine sulfoxide switched from Pla ↔ STA at BL to Pla ↔ VLM trafficking at 6m_FU. Similarly, L-Acetylcarnitine switched from Pla ↔ VLM at BL to VLM ↔ SAA trafficking at 6m_FU.

## 4. Discussion

This study presents a comprehensive system-level investigation of the molecular effects of bariatric surgery-induced weight loss in a cohort of women with obesity, leveraging untargeted metabolomics to examine plasma and tissue-specific responses across multiple clinical samples. Our findings reveal distinct site-dependent metabolite shifts and suggest that metabolic remodelling occurs not only in the circulation but also within regional adipose and muscle tissues. Furthermore, we provide the first analysis of coordinated lipid and polar metabolite trafficking between tissues within a human clinical intervention setting, implicating a systemic realignment of metabolic function following bariatric surgery-induced weight loss.

Partial least squares discriminant analysis (PLS-DA) modelling demonstrated robust separation in metabolite profiles between the time of bariatric surgery at BL and 6m_FU, particularly in SAA and VLM, with strong Q2 values exceeding 0.68. These observations suggesttissue-specific sensitivity to bariatric-induced weight loss, with polar metabolites being more responsive in VLM and peripheral STA tissues, while lipids showed greater shifts in central SAA stores and Pla. These outcomes align with our earlier work indicating depot-specific roles of adipose tissue in metabolic regulation and reinforce the concept that upper body subcutaneous (possibly mixed with ‘overspill’ intradermal lipid) and lower body (subcutaneous) peripheral depots do not respond uniformly to weight reduction [18,33].

Prior to bariatric surgery BL, participants underwent a 2–6-week total meal replacement VLED programme aimed at helping participants lose ~10% of their initial body weight, reduce liver size, and aiding in the pre- and post-operative metabolic and clinical outcomes [7,8]. Pla samples obtained during CID1/Enrol, before starting the VLED programme, also showed a strong separation of both lipids and polar metabolites relative to CID2/BL. Furthermore, pre-operative VLED weight loss also significantly reduced Pla Valine (Val), Isoleucine (Ile), Leucine (Leu), Phenylalanine (Phe), and Tyrosine (Tyr) levels over the 2–6-weeks. It has been well documented throughout the literature and previously reported in our own clinical trials that circulating BCAAs and AAAs are negatively correlated with insulin resistance and metabolic dysfunction while having positive correlations with weight loss [34,35,36]. Also, plasma amino acids are a group of metabolites that undergo significant changes following bariatric surgery procedures [37,38]. There is, however, poor agreement as to surgery-related differences in individual amino acids, with even less knowledge of tissue metabolite responses [39,40]. While the Pla results from the pre-operative VLED programme indicate healthier metrics for participants going into surgery, they also suggest an important early and rapid onset of the body’s metabolism and catabolic processes independent of later bariatric-driven outcomes.

Interestingly, while the most significant fold change for all circulating BCAA and AAA levels was unsurprisingly between Enrol and 6m_FU (*p* < 0.01), the decrease in Pla amino acids during post-surgery weight loss (BL to 6m_FU) was insignificant, except for Val, regardless of the 2-fold increase in weight loss between the two timepoint comparisons (−9.2 ± 4.2 kg. vs. −24.5 ± 8.3 kg, respectively). Previous trials of SG and RYGB procedures have shown a significant decrease in a wide number of BCAAs, AAAs, alanine, aspartate, glutamate, leucine, and isoleucine, among others [38,41]. Interestingly, SAA tissue was the only biopsy site showing significant post-surgical reductions in Ile, Leu, Phe, and Tyr levels, suggesting a depot-specific role in amino acid metabolism. This may indicate a reprogramming of SAA metabolism in response to the reduced systemic energy load, potentially reflecting improved metabolic flexibility or altered amino acid catabolism.

The most novel contribution of this clinical metabolomics bariatric follow-up study was utilising the LipidTA tool to perform a Jaccard–Tanimoto similarity-based traffic analysis, which has been shown to elucidate feature trafficking changes between compartments within a network [24,25,29,32]. We calculated a Jaccard–Tanimoto coefficient (JTC) and t-test *p*-value (*p*) for determining directional metabolite flow, including specific lipid and polar metabolite species shuttled between Pla circulation and SAA, STA, and VLM tissue compartments, indicating active inter-organ communication. These observations suggest that weight loss induces not just static concentration changes but a coordinated redistribution of metabolic substrates across the body. For instance, many of the lipids identified through the 6-month bariatric follow-up (BL vs. 6m_FU) LTA increased in compartment trafficking at 6m_FU. While there were no direct trafficking signals detected between SAA and STA compartments, our metrics indicate trafficking through Pla ↔ VLM channels to change access to different adipose depots (VLM ↔ STA ↔ SAA). Of note, the majority of the trafficked lipid species contained poly-unsaturated acyl chains of odd length acyl chains, with an abundance of very-long-chain omega-3 fatty acids, indicating a shuffling of microbiome-associated metabolites, which are less frequent than lipid species containing acyl chains coming from eukaryotic de novo lipogenesis [42,43,44,45].

The trafficking in distinct lipid species to adapt to changes in global metabolic processing signals can be further supported through features identified in the polar metabolite traffic analysis (MTA). In particular, the amino acid derivatives acetyl-L-carnitine and trimethyl-L-lysine were found to move primarily between Pla ↔ VLM compartments at BL, and by 6m_FU had begun trafficking to adipose depots (SAA and STA), suggesting a functional support of fatty acid oxidation capabilities by mitochondrial β-oxidation, potentially enhancing site-specific energy expenditure post-operatively and post-weight loss [46]. Similarly, 2-aminoadipic acid, an intermediate in lysine catabolism, was found to increase its trafficking network to include both adipose depots at 6m_FU has been well characterised in the literature as reducing adiposity and increasing site-specific energy expenditure by regulating key adipocyte thermogenesis and glucose metabolism pathways, providing protective signals against obesity and T2D [47,48]. In conjunction with our results, asymmetric dimethylarginine (ADMA, an inhibitor of nitric oxide synthase) has consistently been reported to decrease in venous circulation following bariatric surgery, thereby signalling improved endothelial function, which in turn may result in decreased blood pressure and a decreased risk of CV events [49,50]. In our data, the calculated error-normalised fold change (ENFC) for ADMA decreased in circulating Pla (−1%), but via traffic flow, increased within VLM (+2%) and STA (+8%) compartments at 6m_FU. This indicates that ADMA may not globally decrease after bariatric surgery; rather, the decrease observed in Pla may represent movement to other tissues after bariatric surgery-induced weight loss.

In addition to core energy-related pathways, several MTA-identified polar metabolites have previously been associated with metabolic regulation and weight loss phenotypes. Guanidinoacetic acid, a precursor in creatine biosynthesis, has demonstrated an ability to modulate fat mass and improve energy efficiency in animal models, showing promise as a dietary supplement used to reduce blood glucose via insulinotropic signalling [51]. Trimethylamine N-oxide, a gut microbiota-derived metabolite, has been correlated with total and visceral adiposity in humans [52]. Pla levels were shown to be positively correlated with both VLM and liver in participants with simple obesity (non-T2D) scheduled for bariatric surgery in our previous publication [18]. This is intriguing since the MTA results in the current study indicate that following bariatric surgery-induced weight loss, TMAO is trafficked from Pla to VLM. Many studies have provided contrasting results on the negative influence of TMAO levels on metabolic disorders [53], but more recent reviews have suggested potential benefits depending on the sites of accumulation, with some studies showing that TMAO can ameliorate high-fat-diet-induced adiposity by modulating ER stress responses and improving insulin sensitivity in tissues such as skeletal muscle, liver, and pancreatic β-cells [54,55]. Acetyl carnosine (or N-Acetylcarnosine, NAC) has been associated with increased fat mass in animal studies, potentially promoting fat retention under certain metabolic conditions [56], and metabolites pyroglutamic acid and 4-imidazolone-5-propionate have been shown to be elevated in states of metabolic dysfunction, including diabetes and malnutrition [57,58,59,60]. Together, these findings underscore the significance of weight loss-induced alterations in polar metabolites that may signal metabolic complications or support metabolic recovery and serve as novel therapeutic markers.

Our findings emphasise the value of integrating multi-tissue metabolomics with network-based analyses to uncover the dynamic nature of metabolic adaptation during bariatric interventions. Our approach offers a holistic view of systemic biochemical interactions. Importantly, we show that not all metabolite shifts are reflected in plasma alone, underscoring the need to consider regional tissue contributions when interpreting circulating marker displacements.

Despite the novel insights resulting from our metabolomics traffic analysis of bariatric surgery-induced weight loss, there were some limitations. The study comprised a small cohort of women with obesity, sampled from a multi-ethnic population in Auckland, New Zealand. Although deemed valid through power calculations, the cohort size at 6m_FU was impacted by participant drop-out (lost to follow-up), the inability to collect a complete set of plasma and tissue samples in all participants, and variable sample quality in some participants post-acquisition. Tissue sampling of peripheral depots (SAA, STA, and VLM) was on occasion constrained by the semi-invasive nature of needle biopsy, whilst deeper visceral and hepatic sites could not be accessed without further laparoscopic surgery. The rate of weight loss following bariatric surgery is rapid and, of course importantly, may not be linear; hence, multiple FU samples would have been informative. Our study was not able to be further expanded to include multiple (e.g., 0, 2, 4, and 6 months) timepoints, which constrained the scope of metabolic flux assessments post-surgery. Also, it would be of interest to undertake an independent validation study to confirm outcomes from this discovery cohort.

While the primary goal of bariatric surgery is to induce weight loss by means of fat depot catabolism, studies have shown participants to be prone to adverse muscle and bone mineral loss over 24 months post-surgery [61]. Rapid body weight loss results in both lean and adipose mass loss, with increased physical activity protecting against skeletal muscle loss and sarcopenia. This may be more important in older women since protein synthesis decreases, and catabolism increases, with age and less important in middle-aged women such as those in our current study (mean age 43 years), although maintaining skeletal muscle mass even in middle age remains important to ensure ‘healthy ageing’ and maintenance of mobility into old age. Our current data does indicate that, even in this group of middle-aged women, there was increased movement of lipids and polar metabolites from all tissue types, indicating a global shift towards metabolic catabolism. Some participants may also be susceptible to relapse with weight re-gain and metabolic complications [62]. Additional sampling may have quantified flux through these events.

Notably, at least in the short-/medium-term, bariatric surgery-induced weight loss is driven by changes in dietary habits. Decreased energy, macronutrient, and micronutrient intake may occur, alongside changes in physical activity. The significant drop in nutrient uptake associated with increased satiety, reduced food load, and reduction in digestive enzyme availability requires careful management in these patients, who require ongoing lifestyle advice regarding both diet and physical activity, to optimise long-term outcomes [63,64]. Importantly, changes in diet and exercise are likely to have significantly contributed to the metabolic shifts observed in our current study, alongside those driven by adipose and lean mass changes per se. The VLED prior to surgery (Enrol to B/L) altered several metabolites, and it cannot be ignored that some changes attributed to surgery may include carry-over effects from this acute dietary change plus body weight and adipose mass loss. Of note is the absence of significant change in plasma amino acids from BL to 6m_FU relative to concentration changes from Enrol to BL. We propose that the initial acute decrease had reached a plateau by 6m_FU. These would include BCAAs and AAAs, which were correlated with poor T2D metrics. With a reduction in these AAs, and the knowledge of a reduced dietary intake, we can propose a reduction in essential AAs, which may worsen detrimental effects on muscle tissue mass.

While the Jaccard–Tanimoto analysis offers a unique framework for identifying inter-compartment metabolite movement, it lacks capability for functional validation via isotope tracing or transporter assays and is unable to confirm the directionality or mechanistic basis of the inferred trafficking in participants. Additionally, researchers have more recently begun advocating the use of targeted and semi-targeted metabolomics as the use of untargeted metabolomics yields relative abundance. Absolute quantification of annotated metabolites with informative concentrations in samples may generate additional insightful predictions about an individual’s metabolic flux [65].

Future studies should address these limitations by incorporating larger and more diverse cohorts, extending the follow-up beyond 6 months to assess long-term metabolic adaptations, while monitoring the participant’s diet and physical activity regimes. Functional validation approaches, such as tracer-based metabolomics, would be valuable to confirm dynamic metabolite redistribution across tissues. Inclusion of dietary intake and microbiome profiling may also help explain observed shifts in lipid species linked to microbial metabolism. Ultimately, exploring the prognostic value of specific trafficking patterns, such as shifts in 2-aminoadipic acid or ADMA, may uncover biomarkers predictive of improved metabolic outcomes post-surgery and inform tissue-targeted therapeutic strategies.

## 5. Conclusions

This study demonstrates that bariatric surgery-induced weight loss drives widespread tissue-specific metabolic remodelling, involving not only concentration changes but also inter-tissue metabolite trafficking. Using untargeted metabolomics and Jaccard–Tanimoto-based traffic analysis, we reveal a novel system-level adaptation involving the redistribution of lipids and polar metabolites between plasma, adipose, and muscle compartments. These findings highlight the dynamic and coordinated nature of post-surgical metabolic regulation and support the use of network-based metabolomics to inform clinical strategies for understanding and managing obesity and metabolic disease. Future studies are warranted to explore the prognostic value of these trafficking patterns and their role in long-term metabolic health outcomes.

## Figures and Tables

**Table 1:** Plasma and peripheral tissue samples collected at each clinical investigation day.

	Enrol	BL	6m_FU
Plasma	24 †	28	26 *
SAA	-	19	21
STA	-	25	19
VLM	-	22	18

† Four participants were omitted due to hospital scheduling of an early start of the pre-operative VLED weight loss programme prior to sample collection. * Two participants declined to attend 6m_FU. Enrol, enrolment; BL, baseline (at surgery); 6m_FU, 6-month follow-up; SAA, subcutaneous abdominal adipose; STA, subcutaneous thigh adipose; VLM, *vastus lateralis* muscle.

**Table 2:** Lipids identified as “switch” changing between samples due to bariatric-induced weight loss in LTA.

Number	Lipids
1	SM(d16:0/25:1)
2	SM(d18:2/16:0)
3	SM(d17:1/16:0)
4	PE(16:0p/18:2)
5	PC(16:0e/22:6)
6	PE(18:0/20:3)
7	SM(d20:0/18:1)
8	SM(d16:1/16:0)
9	SM(d22:1/16:0)
10	PE(16:0p/20:5)
11	PG(18:1/18:1)
12	PE(18:0p/20:4)
13	PC(16:0/20:5)
14	Pl(16:0/20:4)
15	PC(18:0/22:6)
16	SM(d16:0/24:2)
17	SM(d16:0/27:2)
18	SM(d18:1/18:0)
19	PC(18:0/18:1)

**Table 3:** Polar metabolites identified as “switch” changing between samples due to bariatric-induced weight loss in MTA.

No.	Polar Metabolites
1	Acetylcarnosine
2	Guanidoacetic acid
3	Guanine
4	4-Imidazolone-5-propionic acid
5	Pyroglutamic acid
6	Trimethylamine N-oxide
7	2-Aminooctanoic acid
8	Asymmetric dimethylarginine
9	N6,N6,N6-Trimethyl-L-lysine
10	Aminoadipic acid
11	Methionine sulfoxide
12	L-Acetylcarnitine

## Data Availability

Metabolomics datasets (lipids and polar metabolites) are available from the corresponding author upon reasonable request.

## Supplementary Materials

The following supporting information can be downloaded at https://www.mdpi.com/article/10.3390/metabo15080525/s1, Supplementary Figure S1: Principle component analysis (PCA) plots showing (a) lipid profiles and (b) polar metabolite profiles from plasma (Pla: green), subcutaneous abdominal adipose (SAA, yellow), subcutaneous thigh adipose (STA, blue), and *vastus lateralis* muscle (VLM, red) samples obtained at baseline (BL) and 6 month follow up (6m_FU). SAA tissue sample collected above the hip. STA tissue sample superficial relative to deeper VLM tissue sample; Supplementary Figure S2: Top VIP features partial-least squares discrimination analysis (PLS-DA) plots showing separation of plasma (Pla) lipids and polar metabolite profiles at enrolment prior to the start of very low energy diet (VLED)-weight loss programme (Enrol, yellow) vs. baseline, time of surgery (BL, red). Models present respective cumulative metrics of variance (R2cum) and prediction (Q2cum). Models determined significant (p ≤ 0.001), unless indicated; Supplementary Table S1: Participant demographics and anthropometry at baseline (B/L) and 6 month follow up (6m_FU); Supplementary Table S2: Pla lipids model: BL vs. 6m_FU PLS-DA top 30 VIP features; Supplementary Table S3: Pla polar metabolites model: BL vs. 6m_FU PLS-DA top 30 VIP features; Supplementary Table S4: SAA lipids model: BL vs. 6m_FU PLS-DA top 30 VIP features; Supplementary Table S5: SAA polar metabolites model: BL vs. 6m_FU PLS-DA top 30 VIP features; Supplementary Table S6: STA lipids model: BL vs. 6m_FU PLS-DA top 30 VIP features; Supplementary Table S7: STA polar metabolites model: BL vs. 6m_FU PLS-DA top 30 VIP features; Supplementary Table S8: VLM lipids model: BL vs. 6m_FU PLS-DA top 30 VIP features; Supplementary Table S9: VLM polar metabolites model: BL vs. 6m_FU PLS-DA top 30 VIP features.

## Abbreviations

The following abbreviations are used in this manuscript:

6m_FU	6-month follow-up
AAA	Aromatic amino acids
ADMA	Asymmetric dimethylarginine
BCAA	Branched chain amino acids
BL	Baseline
BMI	Body mass index
CID	Clinical investigation day
CV	Coefficient of variation
CV-ANOVA	Cross validation–analysis of variance
DSAA	Deep subcutaneous abdominal adipose
ENFC	Error-normalised fold change
Enrol	Enrolment
ER	Endoplasmic reticulum
ESI	Electrospray ionisation
FC	Fold change
IAA	Intraabdominal adipose
Ile	Isoleucine
JTC	Jaccard–Tanimoto coefficient
Leu	Leucine
LTA	Lipid traffic analysis
MTA	Polar metabolite traffic analysis
NAC	N-Acetylcarnosine
PC	Phosphatidylcholine
PCA	Principle component analysis
PE	Phosphatidylethanolamine
Phe	Phenylalanine
PI	Phosphatidylinositol
Pla	Plasma
PLS-DA	Partial least squares discriminant analysis
QC	Quality control
RAM	*Rectus abdominis* muscle
SAA	Subcutaneous abdominal adipose
SM	Sphingomyelin
STA	Subcutaneous thigh adipose
T2D	Type 2 diabetes
TMAO	Trimethylamine N-oxide
Tyr	Tyrosine
UHPLC-MS	Ultra-high-performance liquid chromatography–mass spectrometry
Val	Valine
VIP	Variable of importance in projection
VLED	Very low energy diet
VLM	*Vastus lateralis* muscle
