# Supplementary material for: Site-Specific Trafficking of Lipid and Polar Metabolites in Adipose and Muscle Tissue Reveals the Impact of Bariatric Surgery-Induced Weight Loss: A 6-Month Follow-Up Study
Source: Metabolites. 2025 Aug 2;15(8):525. doi: 10.3390/metabo15080525 (PMC12388112; doi:10.3390/metabo15080525)
Supplement: Supplementary file 1 [file metabolites-15-00525-s001.zip › metabolites-3760089-supplementary.pdf]

## Supplementary material:

# Site specific trafficking of lipid and polar metabolites in adipose and muscle tissue reveals the impact of bariatric surgery-induced weight loss: a 6 month follow up study

Aidan Joblin-Mills, Zhanxuan E. Wu, Garth J.S. Cooper, Ivana R. Sequeira-Bisson, Jennifer Miles-Chan, Anne-Thea McGill, Sally D. Poppitt and Karl Fraser

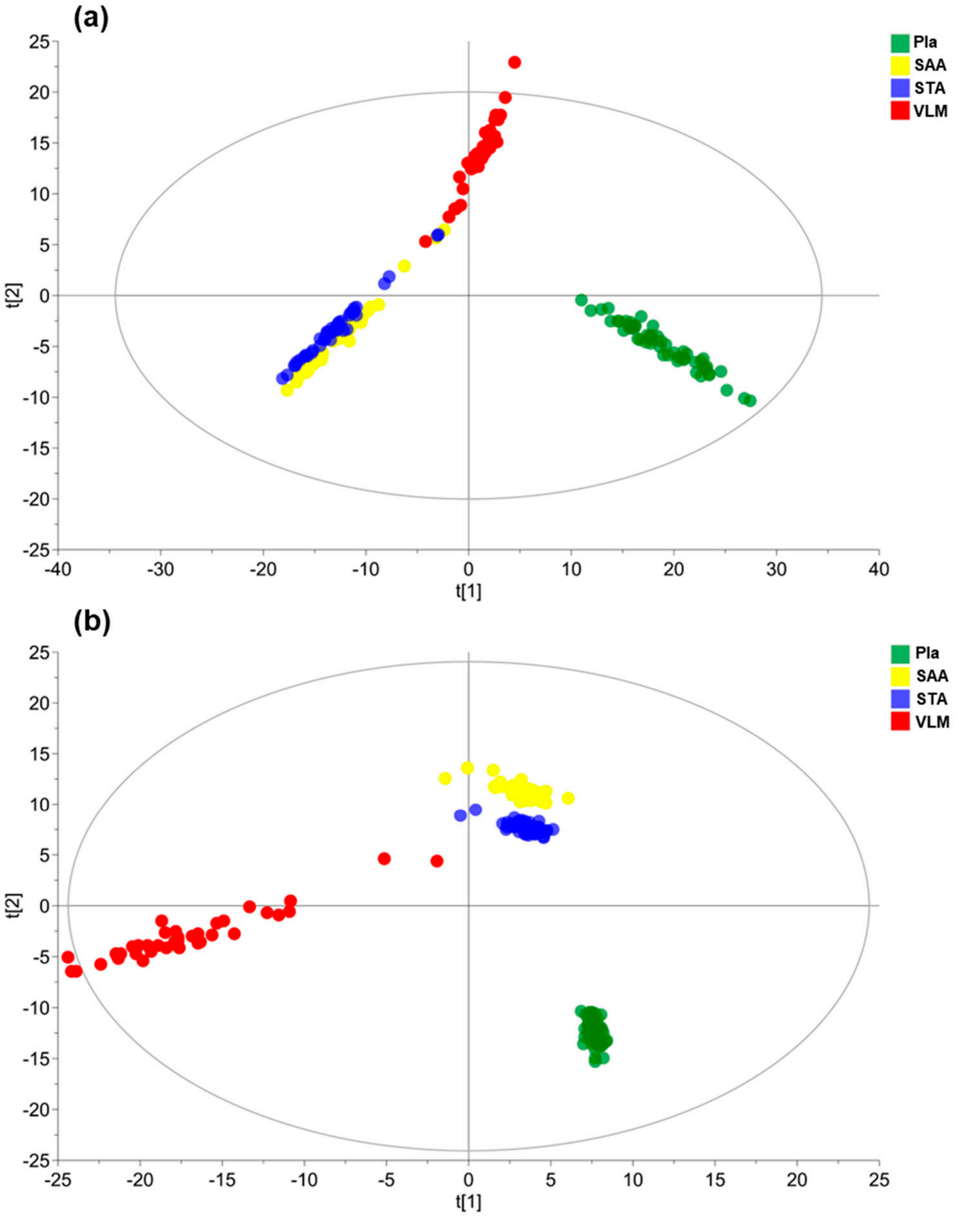


**Supplementary Figure S1.** Principle component analysis (PCA) plots showing **(a)** lipid profiles and **(b)** polar metabolite profiles from plasma (Pla: green), subcutaneous abdominal adipose (SAA, yellow), subcutaneous thigh adipose (STA, blue), and *vastus lateralis* muscle (VLM, red) samples obtained at baseline (BL) and 6 month follow up (6m_FU). SAA tissue sample collected above the hip. STA tissue sample superficial relative to deeper VLM tissue sample.

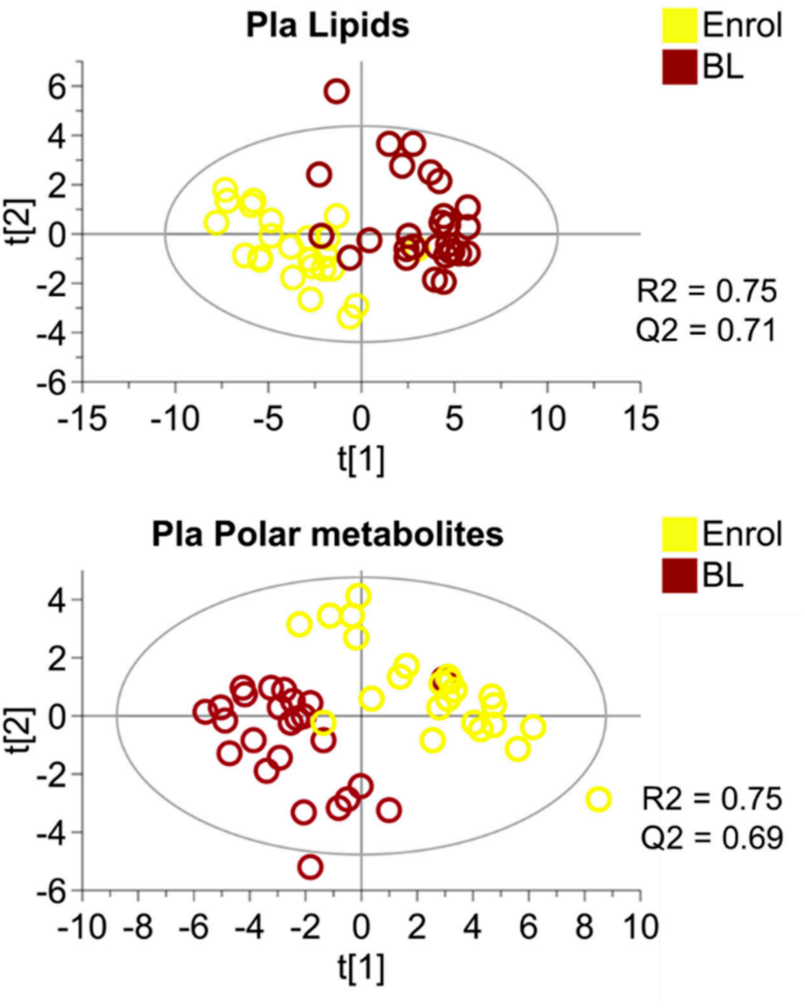


**Supplementary Figure S2:** Top VIP features partial-least squares discrimination analysis (PLS-DA) plots showing separation of plasma (Pla) lipids and polar metabolite profiles at enrolment prior to the start of very low energy diet (VLED)-weight loss programme (Enrol, yellow) vs. baseline, time of surgery (BL, red). Models present respective cumulative metrics of variance (R2cum) and prediction (Q2cum). Models determined significant ($p \leq 0.001$), unless indicated.

**Supplementary Table S1. Participant demographics and anthropometry at baseline (B/L) and 6 month follow up (6m_FU)**

| | Gender | Ethnicity | Age (yrs) | B/L Body weight (kg) | B/L BMI (kg/m$^2$) | 6m_FU Body weight (kg) | 6m_FU BMI (kg/m$^2$) | Bariatric procedure |
|---|---|---|---|---|---|---|---|---|
| 1 | F | Māori | 30 | 137.7 | 54.5 | 103.7 | 41.0 | RYGB |
| 2 | F | Māori | 32 | 122.0 | 44.5 | 90.7 | 33.1 | RYGB |
| 3 | F | Māori | 42 | 130.0 | 44.0 | 100.8 | 34.1 | SG |
| 4 | F | Caucasian | 58 | 119.7 | 44.8 | 96.0 | 35.91 | RYGB |
| 5 | F | Caucasian | 50 | 117.5 | 45.1 | 84.9 | 32.6 | RYGB |
| 6 | F | Māori | 50 | 132.4 | 50.5 | drop out | drop out | SG |
| 7 | F | Caucasian | 45 | 94.7 | 35.6 | 77.4 | 29.1 | SG |
| 8 | F | Asian Chinese | 46 | 80.0 | 31.5 | 68.7 | 27.0 | RYGB |
| 9 | F | Caucasian | 35 | 97.0 | 34.0 | 105.0 | 36.8 | RYGB |
| 10 | F | Hispanic/Indigenous American | 35 | 127.0 | 50.6 | 98.3 | 39.1 | RYGB |
| 11 | F | Caucasian | 50 | 111.8 | 36.3 | 66.7 | 21.7 | RYGB |
| 12 | F | Caucasian | 53 | 137.8 | 46.1 | 111.4 | 37.2 | RYGB |
| 13 | F | Māori | 49 | 135.3 | 49.1 | 107.7 | 39.1 | RYGB |
| 14 | F | Pacific | 43 | 101.4 | 39.6 | 82.5 | 32.2 | RYGB |
| 15 | F | Indian | 40 | 89.9 | 37.1 | 74.8 | 30.9 | SG |
| 16 | F | Indian | 57 | 99.9 | 38.8 | 100.2 | 38.9 | SG |
| 17 | F | Māori/Caucasian | 37 | 136.7 | 40.4 | 94.2 | 27.8 | RYGB |
| 18 | F | Caucasian | 36 | 126.6 | 49.4 | drop out | drop out | SG |
| 19 | F | Pacific/Māori | 46 | 108.5 | 42.3 | 93.9 | 36.6 | SG |
| 20 | F | Caucasian/Indigenous American | 51 | 93.8 | 41.7 | 77.1 | 34.3 | RYGB |
| 21 | F | Indian | 44 | 148.0 | 48.1 | 117.2 | 38.1 | SG |
| 22 | F | Pacific | 40 | 103.0 | 39. | 78.7 | 29.8 | SG |
| 23 | F | NZ European | 49 | 124.0 | 49.1 | 92.2 | 36.5 | SG |
| 24 | F | Māori | 36 | 114.7 | 46.0 | 92.4 | 37.0 | RYGB |
| 25 | F | Caucasian | 48 | 97.3 | 36.9 | 73.3 | 27.8 | RYGB |
| 26 | F | Caucasian | 35 | 111.6 | 43.6 | 85.1 | 33.2 | RYGB |
| 27 | F | Caucasian | 34 | 126.3 | 43.2 | 100.6 | 34.4 | RYGB |
| 28 | F | Caucasian | 58 | 107.8 | 36.9 | 89.4 | 30.6 | SG |

**Supplementary Table S2: Pla lipids model: BL vs. 6m_FU PLS-DA top 30 VIP features**

| Annotation | m/z | RT (min) | Adduct | Pla.VIP[5] | Pla.VIP[5]cvSE × 2.44693 |
|---|---|---|---|---|---|
| PC(16:1p/18:0) | 744.59 | 6.54 | $[M+H]^+$ | 1.19133 | 0.364551 |
| PC(18:2p/17:0) | 756.59 | 6.23 | $[M+H]^+$ | 1.16548 | 0.209576 |
| PE(16:0p/18:2) | 698.51 | 6.17 | $[M-H]^-$ | 1.1638 | 0.542462 |
| PE(18:0p/18:2) | 726.54 | 6.85 | $[M-H]^-$ | 1.15704 | 0.457627 |
| SM(d16:0/20:4) | 725.56 | 5.46 | $[M+H]^+$ | 1.14694 | 0.815246 |
| PC(16:0p/18:2) | 742.58 | 6.01 | $[M+H]^+$ | 1.12184 | 0.393567 |
| PC(18:0p/18:2) | 770.61 | 6.71 | $[M+H]^+$ | 1.10693 | 0.323675 |
| PC(18:0e/18:2) | 772.62 | 6.80 | $[M+H]^+$ | 1.06492 | 0.230673 |
| PS(18:2/21:1) | 826.56 | 5.26 | $[M-H]^-$ | 1.03447 | 0.294847 |
| PS(18:1/21:0) | 830.59 | 6.34 | $[M-H]^-$ | 1.02251 | 0.240729 |
| FA(16:0) | 255.23 | 3.66 | $[M-H]^-$ | 1.01937 | 0.809025 |
| PC(20:0/18:2) | 814.63 | 6.95 | $[M+H]^+$ | 1.01471 | 0.160435 |
| PC(16:0/22:4) | 854.59 | 5.90 | $[M+HCO_2]^-$ | 1.00749 | 0.432169 |
| PC(16:0e/16:1) | 718.57 | 6.55 | $[M+H]^+$ | 0.999753 | 0.337543 |
| PC(18:0/18:1) | 788.62 | 6.95 | $[M+H]^+$ | 0.999112 | 0.282477 |
| PC(17:0/18:2) | 772.59 | 6.00 | $[M+H]^+$ | 0.989908 | 0.3059 |
| PC(18:0/18:2) | 786.60 | 6.38 | $[M+H]^+$ | 0.983704 | 0.240589 |
| PC(16:1/19:0) | 774.60 | 6.52 | $[M+H]^+$ | 0.978876 | 0.518794 |
| SM(d18:0/18:0) | 733.62 | 6.39 | $[M+H]^+$ | 0.97463 | 0.310285 |
| PC(16:0p/20:4) | 766.57 | 5.86 | $[M+H]^+$ | 0.964061 | 0.850136 |
| PC(17:1/18:2) | 770.57 | 5.52 | $[M+H]^+$ | 0.933277 | 0.357719 |
| HexCer(42:1) | 856.69 | 8.35 | $[M+HCO_2]^-$ | 0.926399 | 0.486454 |
| LPC(18:0) | 568.36 | 2.59 | $[M+HCO_2]^-$ | 0.925351 | 0.490403 |
| PC(18:0/16:0) | 762.60 | 6.90 | $[M+H]^+$ | 0.898779 | 0.453496 |
| FA(16:1) | 253.22 | 2.98 | $[M-H]^-$ | 0.880298 | 0.819687 |
| SM(d18:2/18:0) | 773.58 | 5.55 | $[M+HCO_2]^-$ | 0.868293 | 0.511326 |
| SM(d20:1/16:0) | 775.60 | 6.13 | $[M+HCO_2]^-$ | 0.862456 | 0.49609 |
| SM(d16:0/22:4) | 753.59 | 6.13 | $[M+H]^+$ | 0.861944 | 0.56778 |
| SM(d16:1/18:1) | 701.56 | 4.90 | $[M+H]^+$ | 0.808136 | 0.351362 |
| SM(d18:1/18:0) | 731.61 | 6.16 | $[M+H]^+$ | 0.742687 | 0.45776 |

**Supplementary Table S3: Pla polar metabolites model: BL vs. 6m_FU PLS-DA top 30 VIP features**

| Annotation | m/z | RT (min) | Adduct | Pla.VIP[2] | Pla.VIP[2]cvSE × 2.44693 |
|---|---|---|---|---|---|
| Prolylhydroxyproline | 229.12 | 13.5 | M+H+ | 1.42535 | 0.537558 |
| 2-Pyrrolidone-5-carboxylic acid | 130.05 | 6.4 | M+H+ | 1.42478 | 0.490938 |
| L-Arginine | 175.12 | 16.1 | M+H+ | 1.40378 | 0.544386 |
| Phenylacetylglutamine | 265.12 | 6.0 | M+H+ | 1.40365 | 0.539042 |
| Pyroglutamine | 129.07 | 12.7 | M+H+ | 1.24454 | 0.546021 |
| DL-2-Aminooctanoic acid | 160.13 | 9.7 | M+H+ | 1.24413 | 0.551104 |
| Diaminohexanoate | 147.11 | 15.5 | M+H+ | 1.21035 | 0.382899 |
| L-Citrulline | 176.10 | 14.0 | M+H+ | 1.13406 | 0.421502 |
| DL-3-Aminoisobutryic acid | 104.07 | 12.0 | M+H+ | 1.05701 | 0.490165 |
| Trimethylamine N-oxide | 76.08 | 10.3 | M+H+ | 1.04526 | 0.703448 |
| Acetylcarnosine | 269.12 | 12.4 | M+H+ | 1.0448 | 0.76096 |
| Glycocyamine | 118.06 | 13.3 | M+H+ | 0.995885 | 0.269934 |
| Hypoxanthine | 137.05 | 8.5 | M+H+ | 0.99238 | 0.267019 |
| Hydroxy-L-proline | 132.07 | 12.5 | M+H+ | 0.902046 | 0.571073 |
| L-Histidine | 156.08 | 15.7 | M+H+ | 0.896734 | 0.499198 |
| L-Acetylcarnitine | 204.12 | 9.0 | M+H+ | 0.871977 | 0.352561 |
| Citric acid | 215.02 | 12.5 | M+NH4+ | 0.851205 | 0.417766 |
| L-Glutamic gamma-semialdehyde | 132.07 | 14.7 | M+H+ | 0.84489 | 0.377156 |
| Glycine | 76.04 | 13.4 | M+H+ | 0.837122 | 0.69776 |
| NG,NG-Dimethyl-L-arginine | 203.15 | 15.1 | M+H+ | 0.829689 | 0.922823 |
| Glutamylalanine | 219.10 | 12.8 | M+H+ | 0.822857 | 0.844766 |
| Dimethylglycine | 104.07 | 10.6 | M+H+ | 0.816075 | 0.403179 |
| Leucylproline | 229.16 | 9.1 | M+H+ | 0.788257 | 1.03898 |
| sn-Glycero-3-phosphocholine | 258.11 | 13.1 | M+H+ | 0.763631 | 0.56101 |
| Uric acid | 169.04 | 11.2 | M+H+ | 0.752173 | 0.342578 |
| 4-Imidazolone-5-propionic acid | 157.06 | 12.7 | M+H+ | 0.75077 | 0.208572 |
| L-Glutamic acid | 148.06 | 13.5 | M+H+ | 0.73507 | 0.503615 |
| Methionine sulfoxide | 166.05 | 13.0 | M+H+ | 0.727789 | 0.197407 |
| 4-Trimethylammoniobutanoic acid | 146.12 | 10.1 | M+H+ | 0.704832 | 0.472159 |
| N1-Methyl-4-pyridone-3-carboxamide | 153.07 | 3.2 | M+H+ | 0.649808 | 0.877432 |

**Supplementary Table S4: SAA lipids model: BL vs. 6m_FU PLS-DA top 30 VIP features**

| Annotation | m/z | RT (min) | Adduct | SAA.VIP[3] | SAA.VIP[3]cvSE × 2.44693 |
|---|---|---|---|---|---|
| PE(16:0p/18:2) | 698.51 | 6.17 | $[M-H]^-$ | 1.07906 | 0.630555 |
| PE(18:0p/18:2) | 726.54 | 6.85 | $[M-H]^-$ | 1.07462 | 0.472101 |
| SM(d18:2/16:0) | 745.55 | 4.89 | $[M+HCO_2]^-$ | 1.39387 | 0.888111 |
| PS(18:1/21:0) | 830.59 | 6.34 | $[M-H]^-$ | 1.04337 | 0.468278 |
| PS(18:0/23:2) | 856.61 | 6.52 | $[M-H]^-$ | 1.05018 | 0.449539 |
| SM(d18:1/24:0) | 859.69 | 8.26 | $[M+HCO_2]^-$ | 0.985623 | 0.575066 |
| DG(16:0/16:1) | 584.53 | 6.86 | $[M+NH_4]^+$ | 0.868895 | 0.26082 |
| SM(d16:0/18:0) | 705.59 | 5.70 | $[M+H]^+$ | 0.949645 | 0.442997 |
| PC(16:0e/16:1) | 718.57 | 6.55 | $[M+H]^+$ | 1.24979 | 0.509819 |
| PC(18:0/18:2) | 786.60 | 6.38 | $[M+H]^+$ | 0.932133 | 0.351096 |
| PC(18:0/18:1) | 788.62 | 6.95 | $[M+H]^+$ | 0.990925 | 0.422276 |
| TG(12:0/17:1/18:2) | 804.71 | 9.90 | $[M+NH_4]^+$ | 0.861208 | 0.301072 |
| TG(15:0/16:1/16:1) | 806.72 | 10.31 | $[M+NH_4]^+$ | 0.912228 | 0.211869 |
| PC(20:1/18:2) | 812.62 | 6.52 | $[M+H]^+$ | 0.943275 | 0.448554 |
| SM(d16:0/26:1) | 815.70 | 8.26 | $[M+H]^+$ | 0.935108 | 0.520296 |
| TG(18:1/12:0/18:2) | 818.72 | 10.12 | $[M+NH_4]^+$ | 0.881761 | 0.332536 |
| TG(15:0/16:1/18:3) | 830.72 | 9.99 | $[M+NH_4]^+$ | 1.07086 | 0.207719 |
| TG(15:0/16:1/18:2) | 832.74 | 10.35 | $[M+NH_4]^+$ | 1.03949 | 0.177205 |
| TG(15:0/16:0/18:2) | 834.76 | 10.76 | $[M+NH_4]^+$ | 0.962935 | 0.273579 |
| TG(14:0/18:2/18:3) | 842.72 | 9.80 | $[M+NH_4]^+$ | 0.972726 | 0.407979 |
| TG(16:1/16:1/18:2) | 844.74 | 10.19 | $[M+NH_4]^+$ | 0.995179 | 0.28816 |
| TG(16:0/16:1/18:2) | 846.75 | 10.57 | $[M+NH_4]^+$ | 0.963829 | 0.350574 |
| TG(16:1/17:1/18:3) | 856.74 | 10.07 | $[M+NH_4]^+$ | 0.992178 | 0.327928 |
| TG(16:1/17:1/18:2) | 858.76 | 10.41 | $[M+NH_4]^+$ | 0.975276 | 0.346469 |
| TG(16:1/17:1/18:1) | 860.77 | 10.80 | $[M+NH_4]^+$ | 0.955464 | 0.332289 |
| TG(16:1/17:0/18:1) | 862.79 | 11.19 | $[M+NH_4]^+$ | 0.929365 | 0.494984 |
| TG(16:1/18:2/18:3) | 868.74 | 9.91 | $[M+NH_4]^+$ | 0.871354 | 0.307278 |
| TG(16:1/16:1/22:6) | 892.74 | 9.80 | $[M+NH_4]^+$ | 0.868811 | 0.508575 |
| TG(16:0/16:1/22:6) | 894.76 | 10.21 | $[M+NH_4]^+$ | 1.10745 | 0.284762 |
| PI(18:0/20:4) | 904.59 | 5.48 | $[M+NH_4]^+$ | 0.957636 | 0.51239 |

**Supplementary Table S5: SAA polar metabolites model: BL vs. 6m_FU PLS-DA top 30 VIP features**

| Annotation | m/z | RT (min) | Adduct | SAA.VIP[2] | SAA.VIP[2]cvSE × 2.44693 |
|---|---|---|---|---|---|
| Urea | 61.04 | 7.5 | M+H+ | 1.35889 | 0.373913 |
| L-Citrulline | 176.10 | 14.0 | M+H+ | 1.35623 | 0.373222 |
| L-Acetylcarnitine | 204.12 | 9.0 | M+H+ | 1.34934 | 0.373257 |
| L-Arginine | 175.12 | 16.1 | M+H+ | 1.3213 | 0.393562 |
| L-Serine | 106.05 | 13.8 | M+H+ | 1.25231 | 0.736502 |
| L-Lysine | 147.11 | 15.9 | M+H+ | 1.09091 | 0.633106 |
| Taurine | 126.02 | 13.1 | M+H+ | 1.02135 | 0.560871 |
| Potassium | 79.99 | 15.1 | M+ACN+H | 0.988609 | 0.885387 |
| L-Threonine | 120.07 | 13.0 | M+H+ | 0.985504 | 0.203788 |
| L-Proline | 116.07 | 11.1 | M+H+ | 0.981514 | 0.144541 |
| L-Leucine | 132.10 | 10.5 | M+H+ | 0.96648 | 0.209575 |
| L-Alanine | 90.06 | 12.7 | M+H+ | 0.959203 | 0.133743 |
| L-Asparagine | 133.06 | 13.8 | M+H+ | 0.957628 | 1.39316 |
| Glycine | 76.04 | 13.4 | M+H+ | 0.936572 | 0.138998 |
| L-Histidine | 156.08 | 15.7 | M+H+ | 0.929098 | 0.130553 |
| L-Tyrosine | 182.08 | 11.8 | M+H+ | 0.928182 | 0.136449 |
| L-Isoleucine | 132.10 | 10.2 | M+H+ | 0.907936 | 0.100865 |
| L-Aspartic acid | 134.05 | 14.2 | M+H+ | 0.907852 | 0.118534 |
| L-Creatinine | 114.07 | 11.0 | M+H+ | 0.903222 | 0.519842 |
| L-Glutamine | 147.08 | 13.5 | M+H+ | 0.901635 | 0.371041 |
| L-Phenylalanine | 166.09 | 10.2 | M+H+ | 0.894357 | 0.137658 |
| L-Methionine | 150.06 | 10.8 | M+H+ | 0.888614 | 0.150264 |
| L-Glutamic acid | 148.06 | 13.5 | M+H+ | 0.8858 | 0.220328 |
| Methionine sulfoxide | 166.05 | 13.0 | M+H+ | 0.880695 | 0.0659438 |
| Hypoxanthine | 137.05 | 8.5 | M+H+ | 0.872199 | 0.0836129 |
| 4-Trimethylammoniobutanoic acid | 146.12 | 10.1 | M+H+ | 0.868631 | 0.136172 |
| Chlorate | 122.93 | 15.1 | M+K | 0.848882 | 0.166689 |
| Phosphorylethanolamine | 142.03 | 14.5 | M+H+ | 0.843791 | 0.127431 |
| Pyroglutamine | 129.07 | 12.7 | M+H+ | 0.803787 | 0.164757 |
| Nicotinamide | 123.06 | 2.7 | M+H+ | 0.799973 | 0.122199 |

**Supplementary Table S6: STA lipids model: BL vs. 6m_FU PLS-DA top 30 VIP features**

| Annotation | m/z | RT (min) | Adduct | STA.VIP[3] | STA.VIP[3]cvSE × 2.44693 |
|---|---|---|---|---|---|
| PE(16:0p/20:4) | 722.51 | 6.0 | $[M-H]^-$ | 0.881936 | 0.76071 |
| SM(d18:2/16:0) | 745.55 | 4.9 | $[M+HCO_2]^-$ | 1.09397 | 0.292818 |
| SM(d18:2/18:0) | 773.58 | 5.6 | $[M+HCO_2]^-$ | 0.891288 | 0.534926 |
| PS(18:0/20:4) | 810.53 | 5.7 | $[M-H]^-$ | 1.25102 | 0.626772 |
| SM(d22:0/18:2) | 829.64 | 6.9 | $[M+HCO_2]^-$ | 0.89478 | 0.341333 |
| TG(25:1/18:1/18:1) | 1000.93 | 11.8 | $[M+NH_4]^+$ | 0.956658 | 0.19752 |
| TG(26:0/18:1/18:2) | 1014.94 | 12.4 | $[M+NH_4]^+$ | 0.979372 | 0.215092 |
| TG(26:0/18:1/18:1) | 1016.96 | 12.5 | $[M+NH_4]^+$ | 0.983735 | 0.242753 |
| TG(20:3/22:1/22:1) | 1038.94 | 12.3 | $[M+NH_4]^+$ | 0.89761 | 0.676978 |
| TG(22:1/21:1/22:1) | 1056.99 | 12.2 | $[M+NH_4]^+$ | 1.05626 | 0.579987 |
| DG(16:0/16:0) | 586.54 | 7.4 | $[M+NH_4]^+$ | 0.857139 | 0.248652 |
| DG(18:0/20:4) | 662.57 | 7.5 | $[M+NH_4]^+$ | 0.963793 | 0.385186 |
| SM(d16:1/18:1) | 701.56 | 4.9 | $[M+H]^+$ | 0.952245 | 0.358549 |
| PC(16:0e/16:1) | 718.57 | 6.5 | $[M+H]^+$ | 1.23577 | 0.803373 |
| PE(18:1p/18:2) | 726.54 | 6.3 | $[M+H]^+$ | 0.906795 | 0.369647 |
| PC(16:0p/18:2) | 742.58 | 6.0 | $[M+H]^+$ | 0.889538 | 0.534266 |
| PC(18:1/18:2) | 784.58 | 5.8 | $[M+H]^+$ | 1.12879 | 0.486311 |
| PC(18:0/18:2) | 786.60 | 6.4 | $[M+H]^+$ | 1.02266 | 0.283644 |
| PC(18:0/18:1) | 788.62 | 7.0 | $[M+H]^+$ | 0.943542 | 0.434099 |
| PI(18:0/20:4) | 904.59 | 5.5 | $[M+NH_4]^+$ | 1.64978 | 0.743577 |
| TG(20:1/18:1/18:2) | 928.83 | 11.5 | $[M+NH_4]^+$ | 0.874562 | 0.238521 |
| TG(20:1/18:1/18:1) | 930.85 | 11.8 | $[M+NH_4]^+$ | 0.927207 | 0.201491 |
| TG(18:1/18:1/21:3) | 940.83 | 11.3 | $[M+NH_4]^+$ | 0.803751 | 0.332776 |
| TG(18:1/18:1/22:4) | 952.83 | 11.2 | $[M+NH_4]^+$ | 0.826029 | 0.128873 |
| TG(18:0/18:1/22:4) | 954.85 | 11.6 | $[M+NH_4]^+$ | 0.871606 | 0.349057 |
| TG(18:1/18:2/22:1) | 956.86 | 11.8 | $[M+NH_4]^+$ | 0.850676 | 0.284592 |
| TG(18:1/18:1/22:1) | 958.88 | 12.1 | $[M+NH_4]^+$ | 0.998324 | 0.381133 |
| TG(18:1/18:1/23:1) | 972.90 | 12.1 | $[M+NH_4]^+$ | 1.05085 | 0.327096 |
| TG(18:1/20:4/22:1) | 980.86 | 11.6 | $[M+NH_4]^+$ | 0.891082 | 0.170868 |
| TG(18:1/18:2/24:1) | 984.89 | 12.1 | $[M+NH_4]^+$ | 1.06184 | 0.5019 |

**Supplementary Table S7: STA polar metabolites model: BL vs. 6m_FU PLS-DA top 30 VIP features**

| Annotation | m/z | RT (min) | Adduct | STA.VIP[4] | STA.VIP[4]cvSE × 2.44693 |
|---|---|---|---|---|---|
| Pyroglutamine | 129.07 | 12.7 | M+H+ | 1.69905 | 0.781388 |
| Trimethylamine N-oxide | 76.08 | 10.3 | M+H+ | 1.58178 | 0.267811 |
| DL-2-Aminooctanoic acid | 160.13 | 9.7 | M+H+ | 1.58113 | 0.253369 |
| Glycine | 76.04 | 13.4 | M+H+ | 1.5309 | 0.319667 |
| Trigonellinamide (1-Methylnicotinamide) | 137.07 | 11.4 | M+H+ | 1.51012 | 0.250117 |
| L-Phenylalanine | 166.09 | 10.2 | M+H+ | 0.993048 | 0.836925 |
| L-Glutamic acid | 148.06 | 13.5 | M+H+ | 0.970833 | 0.360966 |
| L-Methionine | 150.06 | 10.8 | M+H+ | 0.962447 | 0.367557 |
| Choline | 104.11 | 10.6 | M+H+ | 0.926935 | 0.352159 |
| L-Acetylcarnitine | 204.12 | 9.0 | M+H+ | 0.904883 | 0.382715 |
| N6,N6,N6-Trimethyl-L-lysine | 189.16 | 15.3 | M+H+ | 0.902359 | 0.374785 |
| L-Isoleucine | 132.10 | 10.2 | M+H+ | 0.899323 | 0.904481 |
| L-Alanine | 90.06 | 12.7 | M+H+ | 0.891418 | 0.334669 |
| L-Lysine | 147.11 | 15.9 | M+H+ | 0.86115 | 0.312848 |
| L-Tyrosine | 182.08 | 11.8 | M+H+ | 0.853586 | 0.550161 |
| L-Arginine | 175.12 | 16.1 | M+H+ | 0.852555 | 0.312472 |
| L-Serine | 106.05 | 13.8 | M+H+ | 0.850809 | 0.30035 |
| Hypoxanthine | 137.05 | 8.5 | M+H+ | 0.845449 | 0.485948 |
| L-Proline | 116.07 | 11.1 | M+H+ | 0.830723 | 0.397451 |
| NG,NG-Dimethyl-L-arginine | 203.15 | 15.1 | M+H+ | 0.829551 | 0.386762 |
| L-Threonine | 120.07 | 13.0 | M+H+ | 0.82476 | 0.516541 |
| L-Citrulline | 176.10 | 14.0 | M+H+ | 0.810805 | 0.284494 |
| L-Histidine | 156.08 | 15.7 | M+H+ | 0.805532 | 0.446823 |
| L-Valine | 118.09 | 11.2 | M+H+ | 0.795466 | 0.448743 |
| L-Asparagine | 133.06 | 13.8 | M+H+ | 0.779306 | 0.245753 |
| L-Aspartic acid | 134.05 | 14.2 | M+H+ | 0.718521 | 0.357939 |
| L-2-Aminoadipic acid | 162.08 | 12.8 | M+H+ | 0.688522 | 0.404659 |
| Nicotinamide | 123.06 | 2.7 | M+H+ | 0.682413 | 0.446826 |
| Betaine | 118.09 | 9.5 | M+H+ | 0.663454 | 0.32341 |
| L-Glutamine | 147.08 | 13.5 | M+H+ | 0.632228 | 0.401573 |

**Supplementary Table S8: VLM lipids model: BL vs. 6m_FU PLS-DA top 30 VIP features**

| Annotation | m/z | RT (min) | Adduct | VLM.VIP[2] | VLM.VIP[2]cvSE × 2.44693 |
|---|---|---|---|---|---|
| FA(22:6) | 327.23 | 2.9 | $[M-H]^-$ | 1.48512 | 1.40486 |
| PE(16:0p/18:2) | 698.51 | 6.2 | $[M-H]^-$ | 1.17273 | 0.906084 |
| PE(16:0/18:1) | 716.52 | 6.4 | $[M-H]^-$ | 1.03939 | 0.345898 |
| SM(d16:1/16:0) | 719.53 | 4.8 | $[M+HCO_2]^-$ | 1.02944 | 0.4659 |
| PE(18:0p/18:2) | 726.54 | 6.8 | $[M-H]^-$ | 1.65148 | 1.37951 |
| SM(d17:1/16:0) | 733.55 | 5.1 | $[M+HCO_2]^-$ | 1.00785 | 0.738943 |
| PE(18:0/18:1) | 744.55 | 7.1 | $[M-H]^-$ | 0.991824 | 0.695662 |
| PC(16:0e/18:2) | 788.58 | 6.1 | $[M+HCO_2]^-$ | 1.00932 | 1.55878 |
| PC(18:1p/20:4) | 836.58 | 5.9 | $[M+HCO_2]^-$ | 0.591392 | 1.57481 |
| PC(18:0p/20:4) | 838.59 | 6.0 | $[M+HCO_2]^-$ | 0.608862 | 1.46179 |
| PC(16:0/22:5) | 852.57 | 5.6 | $[M+HCO_2]^-$ | 0.785593 | 0.780764 |
| SM(d18:1/24:1) | 857.67 | 7.5 | $[M+HCO_2]^-$ | 0.971171 | 0.542696 |
| PS(25:5/18:0) | 878.59 | 6.0 | $[M-H]^-$ | 0.709266 | 0.72464 |
| CAR(18:0) | 428.37 | 2.8 | $[M+H]^+$ | 0.944237 | 0.969001 |
| DG(16:1/18:1) | 610.54 | 7.0 | $[M+NH_4]^+$ | 1.00769 | 1.54868 |
| SM(d15:0/18:1) | 689.56 | 5.1 | $[M+H]^+$ | 1.01287 | 0.712125 |
| SM(d16:0/18:1) | 703.58 | 5.5 | $[M+H]^+$ | 0.897913 | 1.07102 |
| PC(16:0/14:0) | 706.54 | 5.5 | $[M+H]^+$ | 1.16399 | 0.744318 |
| SM(d16:0/20:4) | 725.56 | 5.5 | $[M+H]^+$ | 0.907999 | 1.07008 |
| PE(18:1p/18:2) | 726.54 | 6.3 | $[M+H]^+$ | 1.03849 | 1.23657 |
| PC(16:1/18:3) | 754.54 | 5.0 | $[M+H]^+$ | 0.557031 | 1.14571 |
| PC(16:1/18:2) | 756.55 | 5.3 | $[M+H]^+$ | 1.41439 | 1.1596 |
| PC(18:0/16:0) | 762.60 | 6.9 | $[M+H]^+$ | 1.13206 | 0.466659 |
| PC(18:0/18:1) | 788.62 | 7.0 | $[M+H]^+$ | 0.7644 | 0.715423 |
| PC(18:1/20:4) | 808.58 | 5.6 | $[M+H]^+$ | 0.701862 | 0.747512 |
| PC(18:0/20:4) | 810.60 | 6.3 | $[M+H]^+$ | 0.851791 | 0.368615 |
| PC(20:1/18:2) | 812.62 | 6.5 | $[M+H]^+$ | 0.908538 | 0.676272 |
| SM(d16:0/26:2) | 813.69 | 7.5 | $[M+H]^+$ | 0.987691 | 0.488577 |
| PC(20:1/20:4) | 836.62 | 6.2 | $[M+H]^+$ | 0.866898 | 0.761754 |
| TG(16:1/18:2/22:6) | 918.76 | 9.9 | $[M+NH_4]^+$ | 0.897407 | 2.21771 |

**Supplementary Table S9: VLM polar metabolites model: BL vs. 6m_FU PLS-DA top 30 VIP features**

| Annotation | m/z | RT (min) | Adduct | VLM.VIP[2] | VLM.VIP[2]cvSE × 2.44693 |
|---|---|---|---|---|---|
| Leucylproline | 229.16 | 9.1 | M+H+ | 1.58648 | 1.14808 |
| sn-Glycero-3-phosphocholine | 258.11 | 13.1 | M+H+ | 1.48466 | 0.992747 |
| Isoleucylproline | 229.16 | 9.6 | M+H+ | 1.36886 | 0.319339 |
| Dimethylglycine | 104.07 | 10.6 | M+H+ | 1.3403 | 1.41615 |
| Glycyl-Threonine/Alanyl-Serine | 218.12 | 14.4 | M+ACN+H+ | 1.33102 | 0.281543 |
| Phenylacetylglutamine | 265.12 | 6.0 | M+H+ | 1.2404 | 0.553407 |
| Chlorate | 122.93 | 15.1 | M+K | 1.21459 | 0.277546 |
| Potassium | 79.99 | 15.1 | M+ACN+H | 0.940091 | 0.346576 |
| DL-2-Aminooctanoic acid | 160.13 | 9.7 | M+H+ | 0.900615 | 0.346952 |
| L-Glutamic acid | 148.06 | 13.5 | M+H+ | 0.896178 | 0.422749 |
| L-Citrulline | 176.10 | 14.0 | M+H+ | 0.895193 | 0.429175 |
| Pyroglutamine | 129.07 | 12.7 | M+H+ | 0.890655 | 0.403346 |
| 4-Trimethylammoniobutanoic acid | 146.12 | 10.1 | M+H+ | 0.889639 | 0.431569 |
| Nicotinamide | 123.06 | 2.7 | M+H+ | 0.889305 | 0.402312 |
| L-Lysine | 147.11 | 15.9 | M+H+ | 0.888694 | 0.376217 |
| L-Arginine | 175.12 | 16.1 | M+H+ | 0.886693 | 0.41208 |
| L-Carnitine | 162.11 | 10.9 | M+H+ | 0.87895 | 0.427965 |
| L-Glutamine | 147.08 | 13.5 | M+H+ | 0.875681 | 0.36622 |
| Taurine | 126.02 | 13.1 | M+H+ | 0.869595 | 0.451582 |
| Hypoxanthine | 137.05 | 8.5 | M+H+ | 0.868292 | 0.375903 |
| N-Acetyl-D-glucosamine | 244.08 | 12.1 | M+H+ | 0.852457 | 0.456483 |
| L-Alanine | 90.06 | 12.7 | M+H+ | 0.841775 | 0.436649 |
| L-Proline | 116.07 | 11.1 | M+H+ | 0.838278 | 0.482313 |
| L-Aspartic acid | 134.05 | 14.2 | M+H+ | 0.818345 | 0.462931 |
| Acetylcarnosine | 269.12 | 12.4 | M+H+ | 0.816111 | 0.435635 |
| 4-Imidazolone-5-propionic acid | 157.06 | 12.7 | M+H+ | 0.806474 | 0.273726 |
| O-Butanoylcarnitine | 232.16 | 7.9 | M+H+ | 0.792398 | 0.412999 |
| Hexose | 203.05 | 14.2 | M+Na+ | 0.770994 | 0.735266 |
| L-Creatinine | 114.07 | 11.0 | M+H+ | 0.76801 | 0.248392 |
| L-Acetylcarnitine | 204.12 | 9.0 | M+H+ | 0.765831 | 0.447352 |
